# Construction of the first high-density genetic linkage map and identification of seed yield-related QTLs and candidate genes in *Elymus sibiricus,* an important forage grass in Qinghai-Tibet Plateau

**DOI:** 10.1186/s12864-019-6254-4

**Published:** 2019-11-14

**Authors:** Zongyu Zhang, Wengang Xie, Junchao Zhang, Na Wang, Yongqiang Zhao, Yanrong Wang, Shiqie Bai

**Affiliations:** 1 State Key Laboratory of Grassland Agro-ecosystems; Key Laboratory of Grassland Livestock Industry Innovation, Ministry of Agriculture and Rural Affairs; Engineering Research Center of Grassland Industry, Ministry of Education; College of Pastoral Agriculture Science and Technology, Lanzhou University, Lanzhou, 730020 People’s Republic of China; 2 Sichuan Academy of Grassland Sciences, Chengdu, Sichuan 611731 People’s Republic of China

**Keywords:** *Elymus sibiricus*, Seed yield-related traits, High density genetic linkage map, Comparative genome analysis, QTL

## Abstract

**Background:**

*Elymus sibiricus* is an ecologically and economically important perennial, self-pollinated, and allotetraploid (StStHH) grass, widely used for forage production and animal husbandry in Western and Northern China. However, it has low seed yield mainly caused by seed shattering, which makes seed production difficult for this species. The goals of this study were to construct the high-density genetic linkage map, and to identify QTLs and candidate genes for seed-yield related traits.

**Results:**

An F_2_ mapping population of 200 individuals was developed from a cross between single genotype from “Y1005” and “ZhN06”. Specific-locus amplified fragment sequencing (SLAF-seq) was applied to construct the first genetic linkage map. The final genetic map included 1971 markers on the 14 linkage groups (LGs) and was 1866.35 cM in total. The length of each linkage group varied from 87.67 cM (LG7) to 183.45 cM (LG1), with an average distance of 1.66 cM between adjacent markers. The marker sequences of *E. sibiricus* were compared to two grass genomes and showed 1556 (79%) markers mapped to wheat, 1380 (70%) to barley. Phenotypic data of eight seed-related traits (2016–2018) were used for QTL identification. A total of 29 QTLs were detected for eight seed-related traits on 14 linkage groups, of which 16 QTLs could be consistently detected for two or three years. A total of 6 QTLs were associated with seed shattering. Based on annotation with wheat and barley genome and transcriptome data of abscission zone in *E. sibiricus*, we identified 30 candidate genes for seed shattering, of which 15, 7, 6 and 2 genes were involved in plant hormone signal transcription, transcription factor, hydrolase activity and lignin biosynthetic pathway, respectively.

**Conclusion:**

This study constructed the first high-density genetic linkage map and identified QTLs and candidate genes for seed-related traits in *E. sibiricus*. Results of this study will not only serve as genome-wide resources for gene/QTL fine mapping, but also provide a genetic framework for anchoring sequence scaffolds on chromosomes in future genome sequence assembly of *E. sibiricus*.

## Background

The tribe Triticeae (Poaceae) includes several major cereal crops (wheat, barley, and rye) and many ecologically and economically important forage grasses [1]*. Elymus* L. is the largest genus in the Triticeae, which comprises about 150 polyploid perennial grass species widely distributed worldwide [2]. Asia is the most important center of origin where approximately 80 *Elymus* species were found [3]*.* Many *Elymus* species are closely related to wheat and barley, and may thus serve as potential gene pool for the improvement of stress tolerance (cold, drought and disease) and other important agronomic traits [4]. *Elymus sibiricus* (Siberian wild rye), which is indigenous to northern Asia, is an important perennial, cold-season and self-pollinated forage grass of the genus *Elymus* [5]. Based on the cytogenetic analysis, *E. sibiricus* is allotetraploid species, containing St and H genomes. The St genome is derived from *Pseudoroegneria spicata* (Pursh) A. Löve, and the H genome is derived from the genus *Hordeum* [6]. *Elymus sibiricus* is widely grown and used for forage production and grassland eco-engineering in the Qinghai-Tibet Plateau region of China, owing to its good forage quality, drought and cold tolerance, and excellent adaptability to local special environments [7, 8]. Despite *E. sibiricus* has various agricultural uses and economically importance, its serious seed shattering makes seed production difficult for this species. For cereal crops and forage grasses, seed yield is affected by many seed yield-related traits, such as spike length, seed width, floret number per spike, 1000-seed weight, and seed shattering, among which seed shattering is a major cause of yield loss [9]. Previous study showed that serious seed shattering may result in up to 80% seed yield losses if harvesting is delayed [10]. As a result, selection for high seed retention and genetic improvement of seed shattering are important breeding objectives for this species. Several major quantitative trait loci (QTLs) and genes for seed shattering have been reported in cereal crops like rice, wheat, barley, maize and sorghum, and a few forage grasses. For example, in rice, *SH4* [11], *qSH1* [12], *OsCPL1* [13], *SHAT1* [14], and *SH5* [15] were identified as major genes for seed shattering, their functions and interactions in regulating abscission layers formation and development were also revealed. In addition, in hybrid *Leymus* (Triticeae) Wildryes, a major-effect QTL for seed retention was identified on linkage group (LG) 6a, which aligns to other seed shattering QTLs in American wildrice, *Zea* and *Triticum* [16]. Together, these studies indicate the presence of QTLs and genes with large effects on seed shattering, and the potential to understand which QTLs or genes play a role in regulating seed shattering.

The availability of genetic map makes feasible the identification of genes for monogenic traits or major loci for quantitative traits, it also provides an important basis for the study of genome structure and evolution [17]. It is particularly important for future positional gene cloning, marker-assisted selection, and comparative genome analysis [18]. The utility of genetic linkage map depends on the types and number of markers used [19]. High-density linkage map lays a foundation for genome assembly and fine mapping of quantitative trait loci (QTL) [20]. To date, several molecular marker systems have been used for the construction of genetic linkage map, including amplified fragment length polymorphism (AFLP) [21], restriction fragment length polymorphisms (RFLP) [22], random amplified polymorphic DNA (RAPD) [23], simple sequence repeat (SSR) [24], sequence-related amplified polymorphism (SRAP) [25], and single-nucleotide polymorphism (SNP) [26]. Among these markers, SNP marker is considered as the most promising molecular marker for high-density genetic map construction due to their abundant and wide distribution in genome. The advent of massive parallel next-generation sequencing (NGS) technologies could identify and obtain thousands of SNPs at the whole genome level, thus making it possible to construct high-density SNP genetic maps. However, whole-genome sequencing and genotyping large populations are still cost-prohibitive [27]. Reduced representation library sequencing is considered to be one efficient strategy to bring down the cost through genome reduction [28, 29]. For example, restriction site-associated sequencing (RAD-seq) sequences only the DNA fragment with restriction sites, and has been used for large-scale SNP discovery and genetic mapping in many species [30, 31]. As a modified reduced representation sequencing technique, specific-locus amplified fragment sequencing (SLAF-seq) has several distinguishing advantages such as reduced sequencing costs, deep sequencing, marker efficiency optimization through pre-designed reduced representation scheme, and double-barcode method for large populations. It is an efficient method for large-scale De Novo SNP discovery and genotyping of large population [32]. Recently, SLAF-seq has been increasingly used for high-density genetic linkage map construction in several crops [33], forage grasses [20], and animal species [34].

Toward improving the understanding of *E. sibiricus* genome arrangement and the genetic control of seed yield-related traits, we constructed a genetic linkage map and identified QTLs related to seed shattering as well as other seed traits. Two *E. sibiricus* genotypes were selected based on their variation for seed yield-related traits. We applied SLAF-seq to develop thousands of SLAF markers (SLAFs) and construct the first high-density genetic linkage map in *E. sibiricus,* then identified QTLs and candidate genes for eight seed yield-related traits. These results could lay a foundation for future functional genetic dissection of key genes related to seed shattering and other seed traits.

## Results

### Analysis of SLAF-seq and SLAF markers

After SLAF library construction and high-throughput sequencing, 253.25 Gb of raw data containing 1267.20 M reads were generated. The average percentage of Q30 (quality scores of at least 30) bases was 93.03%. The average guanine-cytosine (GC) content was 46.69%. To estimate the validity of library construction, we used *Oryza sativa ssp. japonica* (genome size = 382 M) as control. A total of 901,095 reads with 92.17% Q30 bases and 45.32% GC content were generated (Table 1). The number of reads for male and female parents was 29,809,327 and 65,542,805, respectively. The average number of reads for offspring was 5,859,224.46 with 93.03% Q30 bases and 46.69% GC content. The number of SLAF markers generated for male and female were 232,429 and 326,923, respectively. The average number of SLAF marker in the progeny was 202,120 (Table 2). The average sequencing depth was 31.95-fold and 7.51-fold for parents and each progeny, respectively.

**Table 1:** Summary of SLAF sequencing data

Sample	Total Reads	Total Bases	Q30 (%)	GC (%)
Male parent	29,809,327	5,959,471,566	92.35	46.17
Female parent	65,542,805	13,074,619,072	90.48	47.81
Offspring	5,859,224.46	1,171,094,186	93.03	46.69
Control	901,095	180,184,466	92.17	45.32
Total	1267,197,024	253,252,927,896	93.03	46.69

**Table 2:** Summary of SLAF tag information

Sample	SLAF Number	Total Depth	Average Depth (X)
Male parent	232,429	6,242,468	26.86
Female parent	326,923	12,106,883	37.03
Offspring	202,120	1,518,763	7.51

**Fig. 1:**
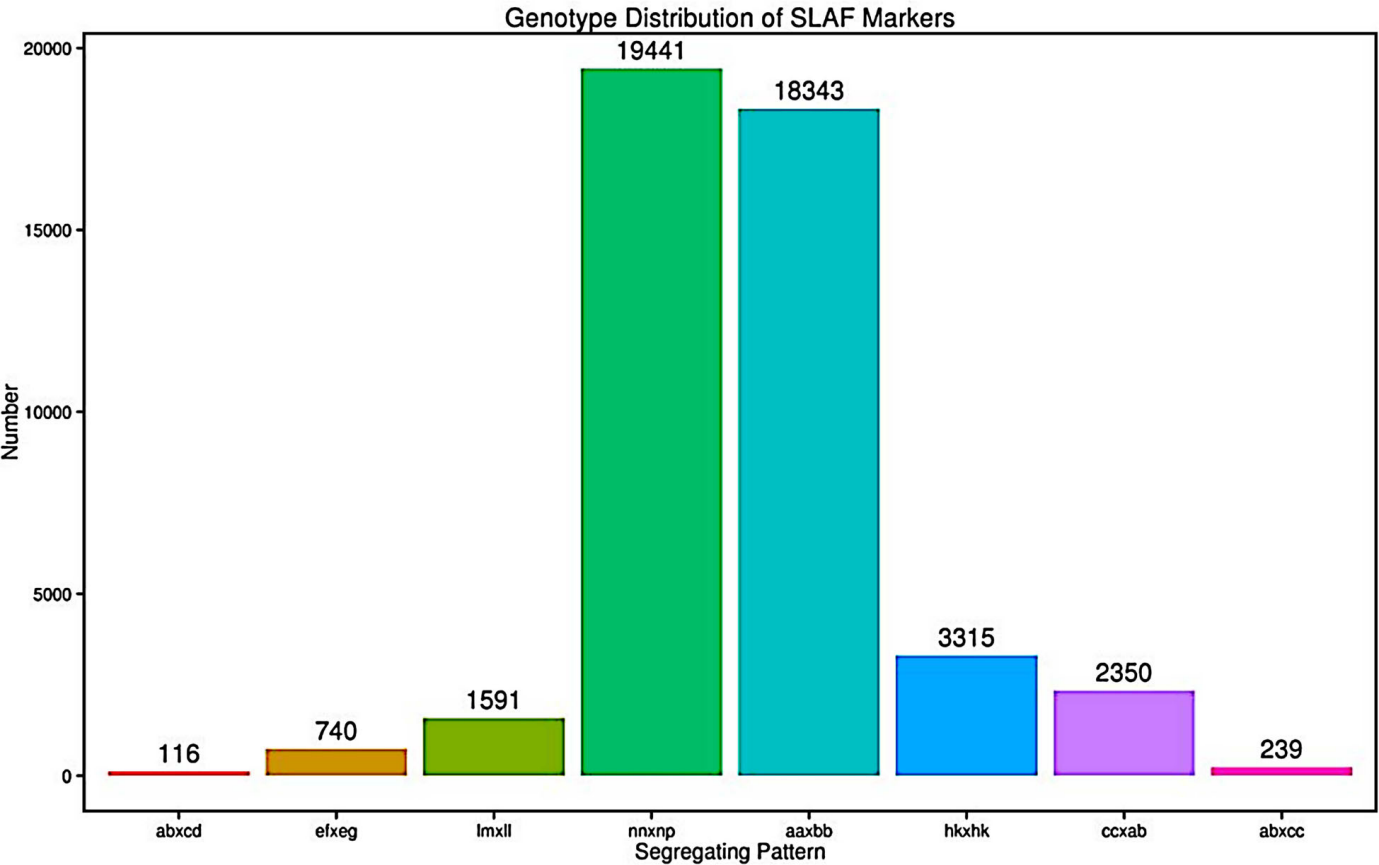
Number of markers for eight segregation patterns

We detected 370,470 SLAF markers, among which 97,387 were polymorphic, 269,579 and 3504 were non-polymorphic (72.77%) and repetitive (0.94%), respectively. Polymorphic markers included mapped biallelic markers and unmapped biallelic markers, monomorphic markers with only one tag in parents were recognized as non-polymorphic markers, mutiallelic markers with tag number larger than 4 in parents were recognized as repetitive markers. Mutiallelic SLAFs which could not be used for recombination rate calculating were removed from further analysis. After filtering the SLAF markers lacking the parent information, 46,135 polymorphic SLAFs were successfully genotyped and further classified into eight segregation patterns (ab×cd, ef × eg, lm × ll, nn × np, aa×bb, hk × hk, cc × ab, ab×cc) (Fig. 1). The mapping population was obtained from the F_1_ hybrid plant of two homozygous parents, therefore, the 18,343 SLAF markers with aa×bb segregation pattern in the F_2_ population were used for genetic map construction.

### Basic characteristics of the genetic maps

**Fig. 2:**
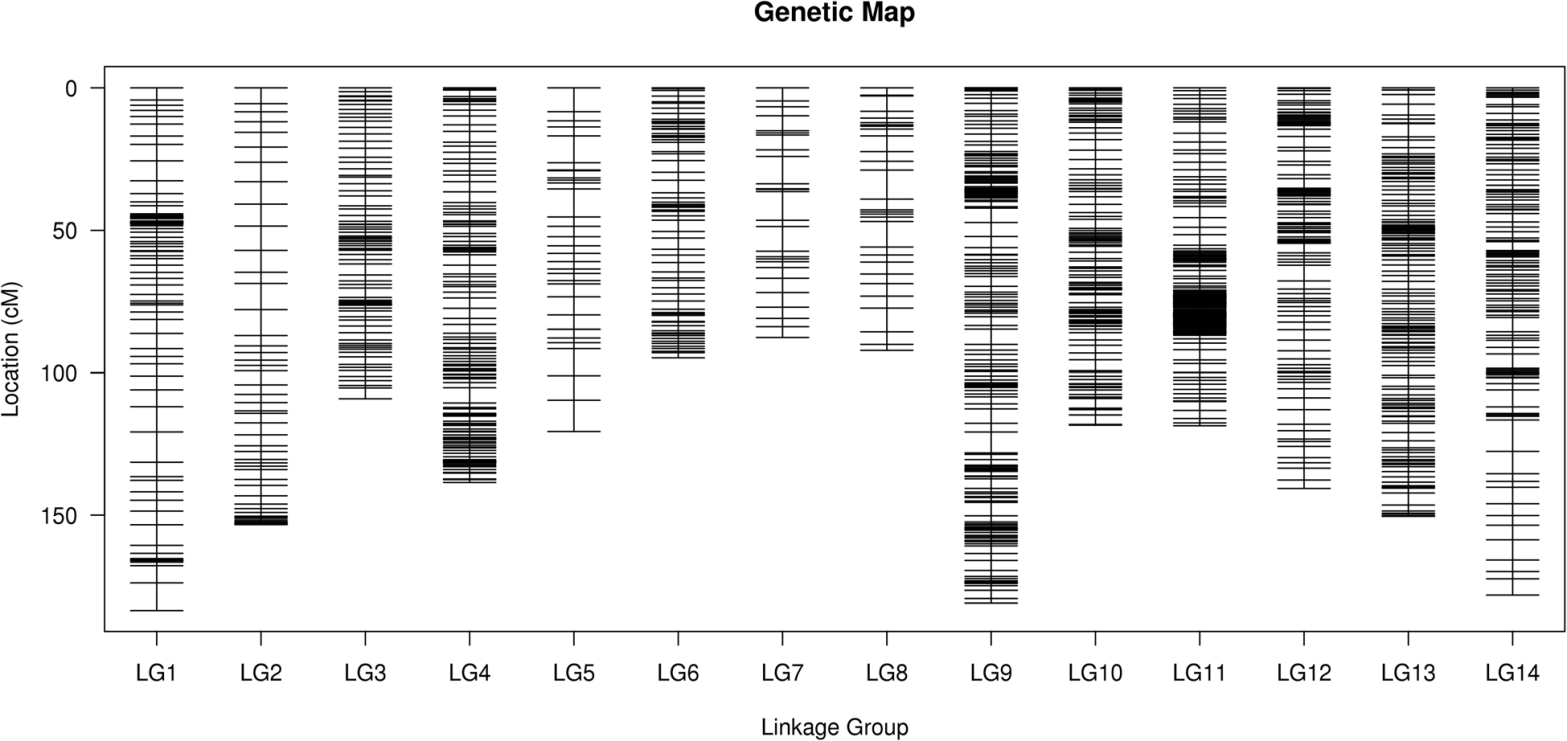
Distribution of SLAF markers on the 14 linkage maps

We further filtered the SLAF markers using four criteria [20]. These SLAF markers that belonging to following four types were removed from mapping construction: SLAF markers from parents with sequencing depth less than 10X; SLAF markers with more than five SNPs; SLAF markers with missing in more than 10% of offspring and segregation-distorted markers (Chi-square, *p* < 0.01). Only the SLAF markers that passed the four-step filtering process were used for constructing a high-quality genetic map. The final map included 1971 markers with 2610 SNP on the 14 linkage groups (LGs) and was 1866.35 cM in length (Fig. 2). The length of each linkage group ranged from 87.67 cM (LG7) to 183.45 cM (LG1), with an average marker density of 1.66 cM between adjacent markers (Table 3). The maximum number of markers (565) were found on LG11, whereas LG8 possessed the minimum number of markers (29) (Additional file 4: Figure S1, Additional file 1: Table S1). The “Gap ≤ 5” value was used to reflect the degree of linkage between each marker, ranging from 73.08 to 100%, with an average of 92.09%. The largest gap on this map was 11.03 cM located in LG14. The number of SNP on each linkage group varied from 35 (LG7) to 712 (LG 11), with an average of 186.

**Table 3:** Description of basic characteristics of the 14 linkage maps

Linkage group	Number of markers			Total Distance (cM)	Average Distance (cM)	Max Gap (cM)	Gaps ≤5 cM
	Total	SNP	Trv/Tri				
LG1	90	113	45/68	183.45	2.04	10.66	88.76%
LG2	56	72	25/47	153.22	2.74	9.2	81.82%
LG3	86	109	30/79	109.09	1.27	3.86	100.00%
LG4	165	229	81/148	138.54	0.84	5.37	99.39%
LG5	33	44	15/29	120.6	3.65	11	75.00%
LG6	87	112	33/79	94.81	1.09	4.41	100.00%
LG7	27	35	13/22	87.67	3.25	10.09	73.08%
LG8	29	44	17/27	92.19	3.18	10.22	82.14%
LG9	276	373	117/256	180.8	0.66	7.36	98.55%
LG10	138	181	55/126	118.38	0.86	3.81	100.00%
LG11	565	712	250/462	118.58	0.21	3.96	100.00%
LG12	138	206	62/144	140.63	1.02	5.52	98.54%
LG13	167	232	73/159	150.41	0.9	4.65	100.00%
LG14	114	148	52/96	177.98	1.56	11.03	92.04%
Total	1971	2610	868/1742	1866.35	1.66	11.03	92.09%

SNP type: Trv means transversion; Tri means transition

In total, only 26 markers showed a significant (*p* < 0.05) segregation distortion and were mapped on the final map, accounting for 1.32% of mapped markers (Table 4). Most of the linkage groups (LGs) had segregation distortion markers with the exceptions of LG1, LG3, LG4, LG13, and LG14. The frequencies of distorted markers on LG6 (19.23%) and LG12 (19.23%) were higher than those of the other linkage groups. LG11, which possessed the maximum mapped markers (565 SLAF markers), had the lowest frequency of distorted marker (3.85%).

**Table 4:** Distribution of segregation distortion markers on each linkage group

Linkage group	Number of distorted markers	Male parent	Female parent
LG1	0	0	0
LG2	2	0	2
LG3	0	0	0
LG4	0	0	0
LG5	3	3	0
LG6	5	5	0
LG7	3	3	0
LG8	2	2	0
LG9	3	0	3
LG10	2	0	2
LG11	1	1	0
LG12	5	2	3
LG13	0	0	0
LG14	0	0	0
Total	26	16	10

### Quality evaluation of the genetic map

To evaluate the quality of the genetic map, haplotype mapping and heat mapping were carried out. The haplotype map reflected the double exchange of the population, which is caused by genotyping error, suggesting a possible recombination hotspot. The haplotype maps of each linkage group were developed for the parental controls and 200 offspring using 1971 SLAF markers. The results showed that most of the recombination blocks were distinctly defined. The LGs 9, 10, and 13 had no missing data, while LG 8 had the largest missing data (3.53%), with an average of 0.73%. Most of the LGs were uniformly distributed (Additional file 5: Figure S2). The heat maps were constructed based on the pair-wise recombination value from the 1971 mapped markers to reflect the recombination relationship between mapped markers on each single linkage group (Additional file 6: Figure S3). The results confirmed the order of mapped SLAF markers on each linkage group.

### Phenotypic variation

**Fig. 3:**
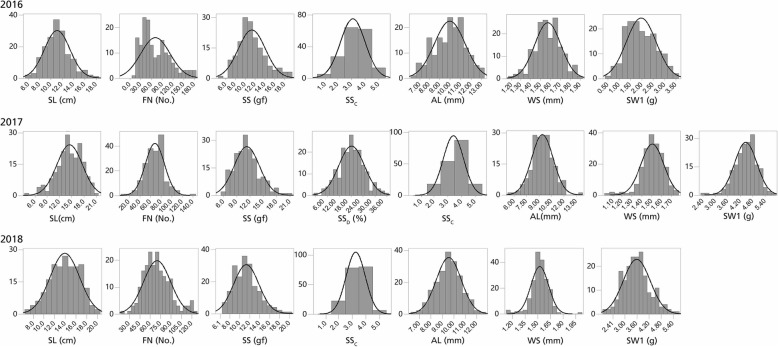
The frequency distribution of eight seed yield-related traits in the F_2_ population. The x-axis shows the ranges of phenotypic traits and the y-axis represents the number of individuals in the F_2_ population

Phenotypic analysis of the parents and F_2_ population revealed significant variations in all eight seed yield-related traits (Table 5, Additional file 2: Table S2). The coefficient of variation (CV) among all traits ranged from 7.24% (WS in 2018) to 58.08% (FN in 2016). We analyzed the correlation between years and traits (Table 6). Our results showed a correlation between phenotypic data detected in different years with exception of WS between 2016 and 2017, and SW1 between 2016 and 2017. For example the correlation for seed shattering (SSc) between 2016 and 2018, 2017 and 2018, 2016 and 2017 were 0.841, 0.783, and 0.360, respectively. Floret number per spike (FN) was significant correlated between 2016 and 2018, 2017 and 2018. Spike length (SL) was significant correlated during 3 years. We calculated the heritability of these traits, all traits had relatively high heritability. The highest heritability (0. 6718) was found for seed shattering (SSc), the lowest heritability (0.4638) was found for floret number per spike (FN). These results were consistent with the correlation analysis between different years. The correlation were found between most traits, for example, awn length (AL) was positively correlated with width of seed (WS), 1000-seed weight (SW1) and spike length (SL). Seed shattering (SS) was positively correlated with floret number per spike (FN). The absolute values of Skewness and Kurtosis for most traits with exception of FN (2017), WS (2017 and 2018), and SW1 (2017) were less than 1 (Table 5). Besides, the normal frequency distributions of eight traits were analyzed and the *P*-value was more than 0.05 except for SL (2017), FN, SS, WS (2017 and 2018) and SW1 (2017) (Fig. 3).

**Table 5:** Descriptive statistics for seed-related traits in the two parents and F_2_ population

Trait	Year	Parents		F_2_ Population							
		Y1005	ZhN06	Max	Min	Mean	SD	CV (%)	Skewness	Kurtosis	Heritability (*h*^2^)
SL (cm)	2016	11.10	14.30	17.87	6.20	11.14	2.25	20.18%	0.439	0.412	0.6227
	2017	15.10	19.26	20.50	4.20	14.50	3.12	21.54%	−0.429	0.018	
	2018	14.31	18.17	20.20	6.57	14.29	2.87	20.11%	−0.247	− 0.359	
FN (No.)	2016	81.67	112.33	183.33	13.00	70.62	41.01	58.08%	0.864	0.098	0.4638
	2017	60.60	108.40	139.60	14.00	68.13	17.99	26.41%	0.138	1.065	
	2018	68.50	109.88	122.50	20.50	69.24	20.27	29.27%	0.535	0.138	
SS (gf)	2016	9.52	12.98	18.80	5.14	11.34	2.75	24.21%	0.651	0.275	0.5235
	2017	9.33	17.61	20.68	5.66	11.30	2.84	25.14%	0.625	0.443	
	2018	9.36	16.84	19.62	6.53	11.61	2.78	23.92%	0.667	0.138	
SS_D_ (%)	2017	27.93	15.55	35.86	0.00	18.19	0.06	35.67%	0.077	0.194	–
SS_C_	2016	1.0	4.0	5.0	1.0	3.11	0.91	29.16%	−0.288	−0.375	0.6718
	2017	1.0	4.0	5.0	1.5	3.41	0.75	21.90%	−0.355	− 0.477	
	2018	1.0	4.0	5.0	1.5	3.27	0.71	21.63%	−0.292	− 0.295	
AL (mm)	2016	12.29	9.88	13.09	6.66	9.95	1.46	14.67%	−0.171	−0.556	0.5281
	2017	11.67	10.35	13.91	5.44	9.41	1.29	13.76%	0.011	0.464	
	2018	11.96	10.29	12.70	6.23	9.54	1.21	12.65%	−0.205	0.128	
WS (mm)	2016	1.60	1.59	1.92	1.19	1.57	0.13	8.42%	−0.113	0.089	0.5086
	2017	1.60	1.30	1.76	1.06	1.51	0.12	7.63%	−0.931	2.367	
	2018	1.58	1.37	2.02	1.15	1.52	0.11	7.24%	−0.397	3.113	
SW1 (g)	2016	3.02	2.32	3.62	0.50	1.97	0.66	33.44%	0.231	−0.635	0.5420
	2017	4.75	3.41	5.70	2.37	4.47	0.54	12.05%	−0.665	1.216	
	2018	3.89	2.87	5.62	1.98	3.62	0.68	18.75%	0.526	0.342	

*SD* standard deviation, *CV* coefficient of variation, *SL* spike length, *FN* floret number per spike, *SS* seed shattering, *SS*_*D*_ seed shattering assessed by dropping from a height, *SS*_*C*_ classification of seed shattering, *AL* awn length, *WS* width of seed, *SW1* 1000 seed weight

**Table 6:** The correlation analysis between three years and eight seed-related traits among F_2_ population

Traits	Year	2016	2017	2018	SL	FN	SS	SS_D_	SS_C_	AL	WS	SW1
SL	2016	1			1							
	2017	0.312**	1		1							
	2018	0.432**	0.981**	1	1							
FN	2016	1			0.646**	1						
	2017	0.182*	1		0.362**	1						
	2018	0.773**	0.736**	1	0.345**	1						
SS	2016	1			0.178*	0.315**	1					
	2017	0.189*	1		0.291**	0.317**	1					
	2018	0.372**	0.978**	1	0.331**	0.275**	1					
SS_D_	2016											
	2017				−0.049	0.052	−0.340**	1				
	2018											
SS_C_	2016	1			−0.142	−0.046	−0.079		1			
	2017	0.360**	1		−0.054	0.168*	0.039	0.064	1			
	2018	0.841**	0.783**	1	−0.074	0.103	0.118		1			
AL	2016	1			0.383**	0.226**	0.113		−0.064	1		
	2017	0.194*	1		0.174*	0.151*	0.108	0.017	0.009	1		
	2018	0.559**	0.920**	1	0.189**	0.133	0.076		−0.063	1		
WS	2016	1			0.470**	0.455**	0.284**		−0.139	0.373**	1	
	2017	0.072	1		0.310**	0.155*	−0.038	0.250**	−0.017	0.288**	1	
	2018	0.510**	0.890**	1	0.224**	0.210**	0.007		−0.134	0.285**	1	
SW1	2016	1			0.144	−0.066	0.069		−0.154	0.202*	0.275**	1
	2017	−0.026	1		0.456**	0.229**	0.018	0.113	0.085	0.325**	0.383**	1
	2018	0.427**	0.684**	1	0.338**	−0.135	0.154*		−0.002	0.150*	0.107	1

* represent significant correlation at 0.05 level, ** represent significant correlation at 0.01 level

### QTL mapping and comparative genome analysis

**Fig. 4:**
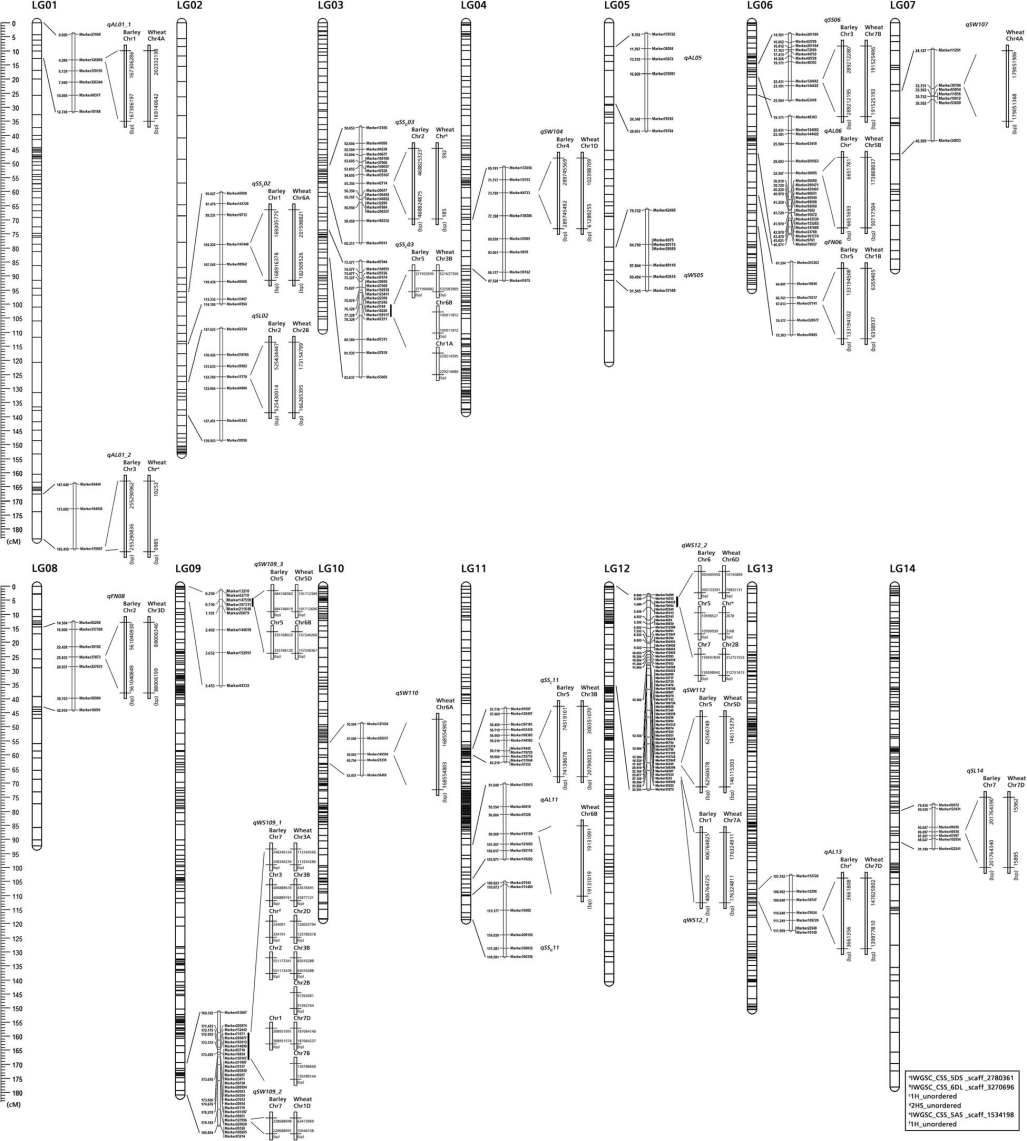
Quantitative trait loci (QTLs) for eight seed yield-related traits. Each QTLs were compared with barley and wheat genomes, respectively

A total of 29 QTLs were detected for eight seed-related traits on 14 linkage groups, of which 3 for spike length (SL), 2 for floret number per spike (FN), 6 for seed shattering (SS, SS_D_ and SSc), 7 for awn length (AL), 3 for width of seed (WS), and 8 for 1000 seed weight (SW1). The LOD and PVE (the percentage of phenotypic variation explained) for all QTLs ranged from 3 to 10.62, 2.17 to 10.85%, respectively (Fig. 4, Table 7). Six QTLs detected for seed shattering explained 2.17 to 9.48% of the phenotypic variation. Among the six QTLs, 1 QTLs were detected on LGs 6 using breaking tensile strength (BTS) data, 2 QTLs were detected on LGs 3 and 11 using seed shattering degree (SS_D_) data, 3 QTLs were detected on LGs 2, 3 and 11 using seed shattering rate (SSc) data. Especially, seed shattering QTLs on LG3 and LG11 could be detected using two methods and at two years (2016 and 2017), respectively. Seven QTLs for awn length (AL) were detected on five linkage groups (LG1, LG5, LG6, LG11 and LG13), among which the QTL on LG1 explained the maximum phenotypic variation of 10.37%. On LG12, a QTL for seed width (WS) was detected and explained the largest phenotypic variation of 10.85% among all QTLs. Moreover, QTLs for awn length (AL) and 1000 seed weight (SW1) were detected on more than five LGs, suggesting a complex genetic mechanism of these traits. A total of 16 QTLs could be consistently detected for two or three years, for example, two QTLs for spike length (SL) on LG14 were detected in 2017 and 2018, two QTLs for seed shattering on LG11 were detected in 2016 and 2017, three QTLs for 1000-seed weight (SW1) on LG9 and three QTLs for awn length (AL) on LG1 were detected for three years.

**Table 7:** Seed-related QTLs detected in F_2_ population of *E. sibiricus* and a comparative genome analysis with Barley and Wheat

Traits	Year	LG	Position (cM)	Markers	LOD	PVE (%)	Barley			Wheat		
							Chromosome	Start	End	Chromosome	Start	End
SL	2016	2	132.789	Marker17770	3.23	6.77	2	525,430,014	525,434,447	2B	166,265,395	173,154,799
	2017	14	86.897	Marker46836	3.90	7.63	7	201,764,340	201,764,390	7D	15,895	15,962
	2018	14	86.897	Marker46836	3.69	8.17	7	201,764,340	201,764,390	7D	15,895	15,962
FN	2016	8	25.833	Marker37873	4.16	8.22	2	561,040,849	561,040,930	3D	88,006,199	88,006,346
	2017	6	67.613	Marker27141	3.87	7.89	5	133,194,102	133,194,508	1B	6,358,937	6,359,405
SS	2016	6	22.431	Marker124682	3.37	8.32	3	289,212,195	289,212,280	7B	191,525,193	191,525,496
SS_D_	2017	3	55.356	Marker42714	3.04	3.50	2	468,824,875	468,825,323	IWGSC_CSS_6DL _scaff_3270696	185	592
		11	117.581	Marker358832	3.74	9.48						
SS_C_	2016	11	59.219	Marker144585	3.13	6.94	5	74,138,678	74,519,101	3B	207,900,333	300,351,070
	2017	2	104.222	Marker147448	3.14	7.27	1	168,916,378	169,305,775	6A	182,509,526	201,508,821
		3	76.328–77.328	Marker5164	3.09	2.17	5	271,933,595	271,994,082	3B	521,637,350	532,583,969
				Marker18220						6B	105,011,872	105,011,972
				Marker159117						1A	229,214,595	229,214,680
AL	2016	1	4.286	Marker126869	5.63	10.37	1	167,306,197	167,306,286	4A	169,140,642	202,332,198
		5	13.755	Marker43872	3.48	5.71						
		6	32.507	Marker36805	3.00	4.70	1H_unordered	6,651,693	6,651,781	5B	50,717,504	173,868,037
		11	99.908	Marker115159	3.10	5.96				6B	19,131,019	19,131,091
	2017	1	183.45	Marker170807	4.12	7.66	3	255,290,836	255,290,962	IWGSC_CSS_5DS _scaff_2780361	9985	10,253
	2018	1	183.45	Marker170807	4.80	9.60	3	255,290,836	255,290,962	IWGSC_CSS_5DS _scaff_2780361	9985	10,253
		13	110.648	Marker78024	3.33	7.73	1H_unordered	3,661,356	3,661,808	7D	139,977,810	147,025,902
WS	2017	5	89.494	Marker83614	3.43	6.34						
		12	27.128	Marker9523	3.59	10.85	1	406,764,725	406,764,825	7A	176,324,811	176,324,911
	2018	12	0–1	Marker74289	10.62	4.63						
				Marker14232			6	305,405,950	305,723,291	6D	14,745,809	79,835,131
				Marker78094			7	150,597,839	150,598,042	2B	312,751,553	312,751,613
				Marker194422			5	10,598,527	10,599,030	IWGSC_CSS_5AS _scaff_1534198	2678	3168
SW1	2016	9	172.925–173.425	Marker71571	3.78	9.10	7	248,346,134	248,346,234	3A	112,164,566	113,254,286
				Marker269877			3	406,889,670	406,889,761	3B	43,676,691	43,677,121
				Marker103013			2HS_unordered	334,091	334,191	2D	120,033,794	125,789,378
				Marker114890			2	551,173,341	551,173,439	3B	65,016,288	65,016,388
				Marker83710						2B	97,262,681	97,262,764
				Marker16854			1	308,951,091	308,951,574	7D	187,064,140	187,064,237
				Marker150167						7B	130,188,660	130,189,144
		10	59.903	Marker146504	3.57	4.75				6A	168,554,803	168,554,905
	2017	4	73.78	Marker64733	4.43	6.24	4	289,745,492	289,745,569	1D	61,289,255	102,398,709
		7	33.751	Marker39194	3.89	8.17				4A	179,051,368	179,051,906
		9	179.183	Marker220020	3.09	5.68						
				Marker127996			7	228,688,948	228,688,991	1D	63,473,969	70,540,158
	2018	7	33.751	Marker39194	3.61	7.72				4A	179,051,368	179,051,906
		9	0.75	Marker147559	3.25	3.26	5	484,748,382	484,748,419	5D	101,712,306	101,712,696
				Marker211648								
				Marker307331			5	335,768,023	335,768,120	6B	157,240,266	157,240,367
		12	17.107	Marker34249	3.56	8.13	5	62,560,678	62,560,749	5D	146,115,303	146,115,379

*LG* Linkage group, *LOD* the logarithm of odds score, *PVE* the percentage of the phenotypic variance explained by individual QTL

**Fig. 5:**
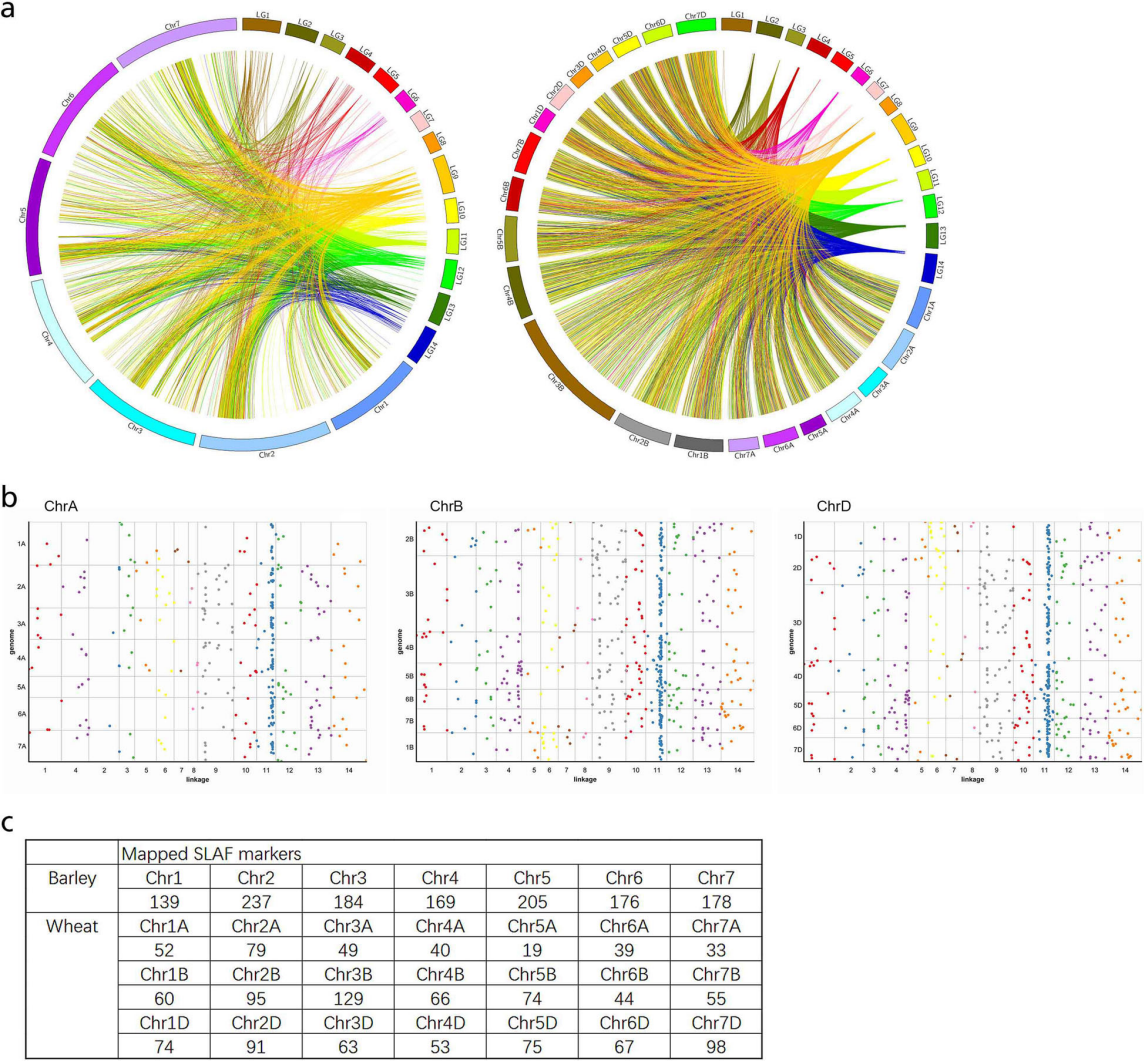
Comparative genome analysis between E. sibiricus and other two grass species. **a**, circos plot showing linear relationship between E. sibiricus with barley (left) and wheat (right); **b**, colinear graph between E. sibiricus and three subgenomes of wheat (A, B and D); **c**, the number of matching markers on ChrA, ChrB and ChrD

The 1971 mapped SLAF markers generated from *E. sibiricus* were compared with the genome sequences of wheat and barley. The Circos plot and Colinear graph was constructed to show the linear relationships between *E. sibiricus* and wheat and barley*,* illustrating a corresponding relationship between the mapped markers and their genomic locations (Fig. 5). The numbers of matching markers between *E. sibiricus* and each species were 1556 (79%) for wheat, 1380 (70%) for barley (Fig. 5a). We further broken down alignments to each subgenome of wheat (A, B and D), the number of matching markers on ChrA, ChrB and ChrD was 311, 523 and 521, respectively (Fig. 5b and c). The largest number of matching markers was found on Chr2A (79), CHr3B (129) and Chr7D (98) for each subgenome, respectively. For barely, the number of matching marker on each chromosome ranged from 139 (Chr1) to 237(Chr2), with an average of 184.

**Fig. 6:**
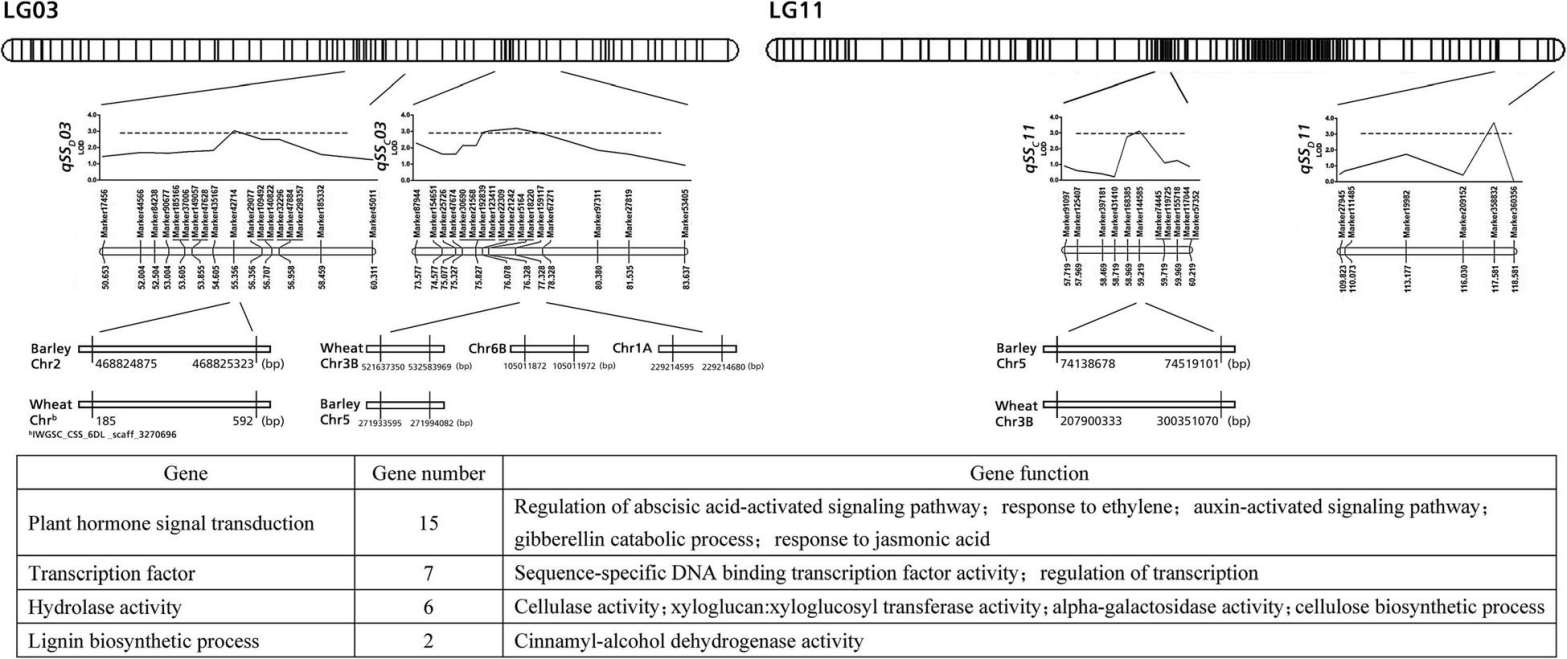
The mapping position and thirty identified candidate genes for seed shattering QTLs on LG3 and LG11

**Fig. 7:**
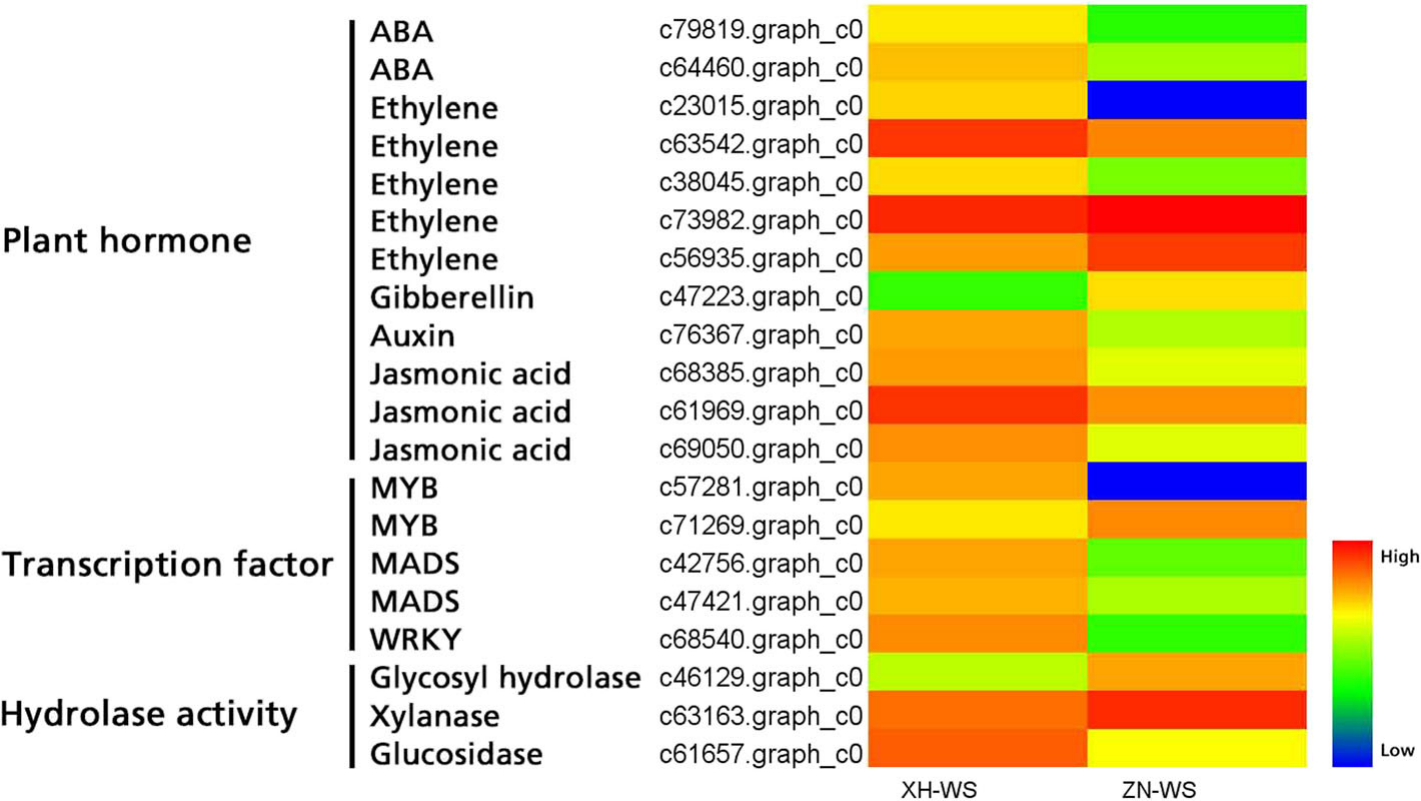
Heatmap diagram of the expression levels of 20 differentially expressed genes involved in seed shattering of two *E. sibiricus* genotypes. XH, high seed shattering genotype, ZN, low seed shattering genotype. WS, 28 days after heading

A total of 43 markers within seed yield-related QTL regions could be identified on barley and wheat chromosomes, of which 3 markers for spike length (SL), 2 markers for floret number per spike (FN), 8 markers for seed shattering (SS, SS_D_ and SS_C_), 7 markers for awn length (AL), 6 markers for seed width (WS), and 17 markers for 1000-seed weight (SW1) (Table 7). These markers were distributed across different chromosomes of each species. For example, 8 markers linked with seed shattering distributed on wheat chromosomes 1A, 3B, 6A, 6B, and 7B, and barley chromosomes 1H, 2H, 3H and 5H. We further identified 30 candidate genes for seed shattering within six QTL regions based on the functional annotation of barley and wheat genomes, of which 15, 7, 6 and 2 genes were involved in plant hormone signal transcription, transcription factor, hydrolase activity and lignin biosynthetic process, respectively (Fig. 6, Additional file 3: Table S3). In particular, among candidate genes for plant hormone, 3 genes were involved in regulation of abscisic acid-activated signaling pathway, 6 genes were involved in ethylene response pathway, 3 genes were involved in auxin-activated signaling pathway, 1 genes for gibberellin, and 1 gene for jasmonic acid. Based on our abscission zone transcriptome data of two *E. sibiricus* genotypes (XH, high seed shattering, ZN, low seed shattering). A total of 20 unigenes involved in plant hormone, transcription factor, and hydrolase activity were predicted from “XH-WS vs ZN-WS”, of which 14 genes were up regulated in high seed shattering genotype XH, 6 genes (2 for ethylene activity, 1 for gibberellin activity, 1 for MYB transcription factor activity, 1 for xylanase activity and 1 for glycosyl hydrolase activity) were up regulated in low seed shattering genotype (Fig. 7). Together, these results suggested these candidate genes might be associated with the regulation of seed shattering in *E. sibiricus*.

## Discussion

### The first linkage map for *E. sibiricus* by next-generation sequencing

*E. sibiricus* breeders have been working to improve economically important traits including yield potential, stress tolerance and persistence. To understand the molecular and genetic mechanisms underlying these important traits, we need some modern methods and tools to effectively identify QTLs and candidate genes involved in these traits. Molecular marker development and genetic linkage map construction are important preliminary basic works for undertaking molecular breeding activities in any crops [35]. Especially, markers strongly related to preferred traits could be used in marker-assisted selection (MAS) to speed up the genetic improvement of desired agronomic traits. To date, a variety of molecular markers including RAPD, SRAP, AFLP and SSRs have been wildly used for linkage map construction in a number of forage grasses such as tall fescue [36], meadow fescue [18], ryegrass [37], orchardgrass [38, 39], alfalfa [40], white clover [41], red clover [42], and *Elymus* wheatgrass [2]. However, the number of mapped markers are limited in many previously reported maps. SLAF sequencing, as a next-generation sequencing technology for genome-wide SNP discovery, has been successfully used for high-density genetic map construction in many plant species including rice [33], cucumber [26], kiwifruit (*Actinidia chinensis*) [43], mei (*Prunus mume*) [44], sesame [45], even in animals such as chicken [46], and white shrimp [34]. However, large scale SNP mining in *E. sibiricus* lagged behind other species due to its large, complex, and nature polyploidy. Here, we used the SLAF-seq to develop and identify 370,470 SLAF markers, of which 97,387 were polymorphic with a polymorphism rate of 26.29%. The polymorphism rate of SLAF markers between the two parents was 26.29%, higher than previous reports in cucumber (9.57%) [26] and sesame (5.12%) [45], suggesting a considerable difference between the two parental genotypes. The linkage map contained 14 linkage groups and spanned 1866.35 cM. Based on SLAF-seq, the map quality of the present genetic maps was similar to previously reported genetic maps for other several species, although, they were the first ones reported for this species. For example, Zhang et al. [45] reported the first high-density genetic map for sesame. A total of 1233 markers were mapped on the 15 linkage groups, with an average marker density of 1.20 cM. In general, these results proved that SLAF-seq is a powerful high-throughput technology for the whole-genome wide SNP discovery and is effective for *E. sibiricus* linkage map construction.

According to our results, the number of SLAF markers on each linkage group varied from 27 (LG7) to 565 (LG 11). Especially, some markers tended to highly cluster in some regions on LG9, LG10 and LG 11. The similar results were found in other plants such as grape [47], sunflower [48], and tree peony [49]. This phenomenon may result from the non-random distribution of mapped markers on linkage group and the uneven recombination rates and marker polymorphism between mapping parents on some chromosomes [50]. Moreover, four gaps larger than 10 cM were located on LG1, LG7, LG8, and LG14. The lack of maker polymorphism and a shortage of marker detection in these regions may have contributed to this finding [51, 52].

### Application of this map for the QTL detection of seed yield-related traits

Like most native grasses, *E. sibiricus* has serious seed shattering which cause large seed yield losses during harvest, making commercial seed production difficult. In this study, Y1005 and ZhN06 were selected and used as parents because they are genetically divergent and have several contrasting seed traits. In the F_2_ mapping population, eight seed yield-related traits showed considerable phenotypic variation. For example, seed shattering in mapping population ranged from 5.14 gf to 18.80 gf during 2016, from 5.66 gf to 20.68 gf during 2017, and from 6.53 gf to 19.62 gf during 2018. Some progenies had lower seed shattering than their parents. Thus, the use of low seed shattering genotype provides QTLs for low seed shattering that could prove robust for breeding lower seed shattering genotypes.

Genetic linkage map allows for comparative genetic studies with other species, provides important information about the genome structure and evolution of a species, and lays a foundation for studying complex and important agronomic traits [53]. The major objective of this study was to reveal the genetic mechanisms controlling seed yield-related traits in *E. sibiricus.* Our comparative genome analysis indicated that *E. sibiricus* was more closely related to wheat and barley. In this study, a total of 8 markers within seed shattering QTL (SS, SS_D_ and SS_C_) regions could be identified on wheat chromosomes 1A, 3B, 6A, 6B, and 7B, and barley chromosomes 1H, 2H, 3H and 5H. In wheat, the *Q* gene on chromosome 5A was identified as a major domestication gene, which encodes an AP2 transcription factor largely responsible for free seed shattering [54]. *TaqSH1*, encoding a BEL1-like protein, was located on the homoeologous group 3 chromosome in wheat. Overexpression of *TaqSH1* gene in transgenic *Arabidopsis* plants could down-regulate several well-known abscission-related genes such as *HAESA* and *KNAT1/6*, suggesting this gene might play as a key upstream regulator for abscission zone development [55]. In barley, the non-brittle rachis trait was controlled by *btr1* and *btr2* genes on chromosome 3H. Further, Pourkheirandish et al. [56] cloned and identified the *Btr1* and *Btr2* genes and elucidated the mechanism underlying the disarticulation of the wild type barley spike.

Based on our comparative genome analysis, marker 5164 and marker 144,585 were on wheat Chr3B, marker 124,682 was on barely Chr 3H. Together, these results highlight the possibility for seed shattering candidate gene identification in *E. sibiricus* based on these genetic maps and the synteny with model grasses. QTLs on other genomic regions may provide new candidate genes for seed shattering in *E. sibiricus*.

### Candidate genes for seed shattering

Plant hormone play an important role in regulating plant growth and development processes. In this study, we identified 15 potential seed shattering candidate genes involved plant hormone including abscisic acid (ABA), ethylene, auxin, gibberellin and jasmonic acid. Abscisic acid regulates many agronomically important development processes and numerous adaptive stress responses in plants [57]. It plays a direct role in abscission of many plant organs such as seed, leaf, flower and fruit [5, 58]. Our previous studies found some ABA-related genes were up-regulated in the abscission zone and suggested these ABA-responsive genes may affect seed shattering [5, 8]. Ethylene is an important regulator of abscission of many plant organ such as seeds, fruit and leaf [58]. Ethylene receptor genes (*ETR1*), the ethylene insensitive mutant of *Arabidopsis,* plays a role in delaying the shedding of floral parts [59]. In our previous study, we reported that 5 *ETR1* gene were up-regulated in abscission zone in *Elymus nutans*, suggesting the roles of *ETR* genes in regulating abscission [60]. Ethylene-responsive factors (*ERF*) also regulate abscission of plant organs, including floral organs, leaf and seed. In tomato (*Solanum lycopersicum*) Ethylene-responsive factor 52 (*SIERF52*) is specifically expressed in pedicel abscssion zones. When the expression of *SIERF52* was suppressed in transgenic plants flower abscission will be significantly delayed compared with wild type [61]. Our transcriptome analysis showed 4 ethylene response factor genes were up-regulated in the abscission zone of high seed shattering genotype, suggesting their roles in promoting seed shattering. Jasmonic acid (JA) is an important regulator of plant growth, development and defense. It had good abscission-promoting effect and positively promoted the abscission of bean petiole expiants in the dark and light without enhancing ethylene production in bean petiole expiants [62]. Similar results were reported in our study, we found three genes involved in JA-mediated signaling pathway were up-regulated in abscission zone of high seed shattering genotype XH-WS. However, it is difficult to identify which hormone or gene is key factor for regulating abscission process, as plant organ abscission is a complex and highly coordinated process involving multiple gene expressions in plant hormone signing pathway. A balance and interaction of these genes may have contributed to seed shattering in *E. sibiricus*.

Transcription factors play an important role in the signal transduction pathways. In this study we identified 7 transcription factor genes, such as MYB, MADS-box and WRKY genes. According to previous study in major crops. Many well-known genes for seed shattering are transcription factors genes like *SH4* [11] and *qSH1* [12]*, STK* [63]*.* In rice, *SH4* is a major QTL for seed shattering, which encodes a MBY3 transcription factor [11]. And many MYB proteins are critical components of multiple hormone-mediated transcriptional regulatory, including ethylene, abscission acid and auxin, which act as important regulators of plant organ abscission [64]. In our previous study, we identified 14 MYB genes up-regulated in the abscission zone in *E. nutans,* indicating their potential roles in regulating the development of abscission zone [60]. In *Arabidopsis*, *STK* is a MADS-box transcription factor gene, which regulates the formation of seed abscission zone [63]. WRKY transcription factors are key components in abscisic acid signaling, and play roles in regulating many plant processes, including seed development, the response to stress, and seed shattering [65]. Therefore, we inferred these candidate transcription factors identified in this study may have the similar functions in the regulation of seed shattering.

Seed shattering is generally caused by the development and degradation of abscission layers that is located in the rachilla just below each seed. Our previous study showed that increased hydrolytic enzymes activity like cellulase and polygalacturonase in abscission zone are highly related to high seed shattering degree in *E. sibiricus* [5]*.* Cellulase is a key hydrolytic enzyme, which plays role in plant cell wall loosening during plant organ abscission [66]. In rice, an endo-1,4,-*β*- glucanase gene named as *OsCel9D*, is an important regulator in modifying cell wall structure and component during abscission, and mutations of this gene will reduce cell elongation and affect cellulose biosynthesis and increase the pectin content, which finally hamper the abscission process in seed shattering [67]. In addition, xyloglucan endotransglucosylase (XTHs) were suggested to loosen plant cell wall through cutting and rejoining the xyloglucans that tether adjacent cellulose miscrofibrils [68]. In this study we identified 2 cellulase genes and 2 XTHs genes, and transcriptome analysis showed that these candidate genes were differently expressed in the abscission zone of two *E. sibiricus* genotypes, indicating these genes may have contributed to seed shattering.

## Conclusions

In general, seed shattering is an important agronomic trait that need improvement during wild grass domestication. Seed shattering is a complex biological process affected by environment factors, cultivation management and multiple changes in the metabolism process, abscission layer cell structure, and functional gene expression level. Previous studies have reported many genes associated with seed shattering, they are involved in hydrolytic enzymes activity, lignin biosynthesis and degradation, plant hormone signaling and response, transcription factors and protein kinase activity [5, 8, 15, 58]. In this study, we constructed the first genetic linkage map and identified seed-related QTLs and candidate genes for seed shattering. Results from this study mostly confirmed previous findings and also reported some new potential candidate genes for seed shattering. More studies are needed to explore their potential roles and functions in seed shattering in the future. In addition, a combination of multiple methods, including genetic mapping and whole-genome association analysis and transcriptome analysis could help us to identify more major loci underlying seed shattering in *E. sibiricus*.

## Materials and methods

### Plant materials and DNA extraction

The mapping population of 200 F_2_ individuals was obtained from self-pollinating a single F_1_ plant, which was developed from a cross between “Y1005” (male parent) and “ZhN06” (female parent). Y1005 and ZhN06 were collected from Sichuan and Gansu provinces, China, respectively. The parents were selected based on a previous evaluation for agronomic traits and genetic diversity [69]. The two parental genotypes are genetically divergent and have several contrasting seed traits, including seed shattering, 1000-seed weight, seed width, spike length, awn length, and floret number per spike.

DNA extraction was carried out with young healthy leaves using the Qiagen DNeasy 96-well procedure (QIAGEN, Valencia, Calif). The quantity and quality of genomic DNA samples were evaluated using the NanoDrop ND1000 spectrophotometer (NanoDrop, Wilmington, DE, USA) and by 0.8% agarose gel electrophoresis.

### Phenotypic evaluation

The F_2_ population of 200 individuals and parents were grown and evaluated in the field of Yuzhong research farm, Lanzhou University, Gansu, China (elevation 1720 m, longitude 103°34′ E, latitude 35°34′ N) for mapping. Plants were spaced 0.5 m within rows and 0.5 m between rows. Phenotypic data for seed yield-related traits were evaluated, including spike length (SL), floret number per spike (FN), seed shattering (SS), awn length (AL), width of seed (WS), and 1000-seed weight (SW1) for three consecutive years (2016–2018). *E. sibiricus* plant has a spike inflorescence containing 15–30 spikelets, each spikelet consists of approximately 5 florets [5]. Three previously reported methods were used to accurately evaluate seed shattering in this study. Breaking tensile strength (BTS) method was firstly used to determine seed shattering. The BTS value was measured upon detachment of seed from the pedicels by pulling, which is negatively related with seed shattering degree. Totally, thirty randomly chosen spikelets from middle part of the spike of each plant were examined at maturity (4 weeks after heading), and their average BTS values were calculated [69]. The second method is that seed shattering degree (SS_D_) was measured following the described procedure by Yao [70] with a minor modification. Three spikes from each plant were released at a height of 1 m and freely fell down onto a hard surface. The seed shattering degree was expressed by a percentage (%) of the number of shattered seeds to the total number of seeds. The third method is that seed shattering rate (SSc) was evaluated at maturity. Based on the number of naturally shattered seeds, seed shattering was rated as follows: 1 (> 80% shattering), 2 (60–80% shattering), 3 (40–60% shattering), 4(20–40% shattering), 5 (< 20% shattering). Other seed traits: SL, FN, AL, WS, and SW1 were measured according to the methods described by Zhang et al. [69]. The descriptive statistics of phenotypic data and the correlation analysis between years and traits were calculated by using SPSS software (SPSS, version 19 for Windows, SPSS Inc., Chicago, IL, USA). Heritability (*h*^2^) for each trait was estimated based on previously reported method: *h*^2^ = *σ*_g_^2^/ (*σ*_g_^2^ + *σ*_gy_^2^/n + *σ*_e_^2^/nr), where *σ*_g_^2^ is the genotypic variance, *σ*_gy_^2^ is the variance caused by the interaction between genotype and year, *σ*_e_^2^ is the error variance, n is the number of years, and r is the number of replications [71].

### SLAF library construction and high-throughput sequencing

SLAF library construction was performed according to the methods described by Sun et al. [32]_._ For maximum SLAF-seq efficiency, a pilot experiment was carried out to establish and optimize the conditions required to avoid repetitive SLAFs, achieve an uniform distribution of SLAFs, and obtain optimal SLAF yield. Briefly, *Hae*III restriction enzymes (New England Biolabs, NEB, USA) was used to digest the genomic DNA of the two parents and F_2_ population. A poly-A was added to the 3′ ends of digested fragments. These digested fragments were then ligated with Dual-index sequencing adaptors, and amplified by PCR. The PCR was carried out in reaction solutions containing the diluted restriction-ligation DNA samples, dNTPs, Q5® High-Fidelity DNA polymerase (NEB) and PCR forward primers 5′- AATGATACGGCGACCACCGA-3′ and reverse primer 5′-CAAGCAGAAGACGGCATACG-3′. The PCR products were then purified using Agencourt AMPure XP beads (Beckman Coulter, High Wycombe, UK) and pooled.

The pooled samples were separated with 2% agarose gel. Fragments of 464–494 bp (with barcodes and adaptors) were excised and purified using a QIAquick gel extraction kit (Qiagen, Hilden, Germany). The obtained SLAFs in the quality-tested library were used for paired-end sequencing on an Illumina HiSeq 2500 sequencing platform (Illumina, San Diego, CA, USA). To check the reliability of testing processes, we used the genome of *Oryza sativa* as a quality control to undergo the same procedures of library construction and sequencing as the *E. sibiricus* mapping population.

### Sequence data analysis and genotyping

SLAF marker identification and genotyping were performed according to the procedures described by Sun et al. [32]. Low-quality reads (with quality score < Q30) were deleted and then the left reads were assigned to the two parents and F_2_ individuals according to the duplex barcodes. The clean reads were obtained after filtering the barcodes and the terminal 5-bp positions from each read. All paired-end reads (200 bp per read) generated from SLAF-seq raw reads were clustered according to their sequence similarity. Sequences with over 90% identity were grouped into one SLAF locus. As *E. sibiricus* is self-pollinated species, an F_2_ population was obtained by self-pollinating the F_1_ plant of a cross between two fully homozygous parents with genotype aa or bb. Therefore, we only used the SLAF markers with aa×bb segregation pattern for genetic map construction.

### Map construction and QTL analysis

HighMap software was used for linkage map construction with four steps: SLAF marker grouping, SLAF marker ordering, genotyping error correction, and genetic map evaluation [72]. The single-linkage clustering algorithm was applied to assign the markers into linkage groups. The modified logarithm of odds (MLOD) score > 5 was set up to partition marker loci into linkage groups (LGs). For comparative genome analysis, we carried out the BLAST search between the mapped SLAF makers and the whole genome sequences of wheat (*Triticum aestivum* L*.*) and barley (*Hordeum vulgare* L*.*) by using an E-value cutoff of 1e-10 and 90% identity cutoff [20]. For QTL identification, the genotypic data of the mapped markers on the linkage map was integrated with the field phenotypic data of eight seed traits. Logarithm of odds (LOD) scores larger than the 5% cutoff value was used to identify significant loci associated with seed traits. The threshold value was determined through 1000 permutation test according to the composite interval mapping (CIM) method from “qtl” package of R. An interval mapping model with LOD scores of 3.0 for potential QTL was used for QTL detection. MapQTL 6.0 [73] was used to estimate the percentage of phenotypic variation and additive effect explained by a QTL for a trait.

### Candidate gene identification

In order to gain an in-depth understanding of the potential functions of these seed yield-related QTLs and identify candidate genes for seed-related traits, the SLAFs within QTL regions were subsequently searched against the *Hordeum vulgare* and Chinese spring wheat reference genome (ftp://ftp.ensemblgenomes.org/pub/plants/release-39/fasta/hordeum_vulgare/dna/; http://www.wheatgenome.org/News/Latest-news/IWGSC-Reference-Sequence-v1.0-browser-now-available-at-URGI) by using the basic local alignment search tool (BLAST). For annotation, the assembled sequences were queried using BLASTX (E-value ≤1e-5) against 7 public databases like the NCBI non-redundant protein sequence (Nr), Gene Ontology (GO), Protein family (Pfam), Cluster of Orthologous Groups (COG), Annotated protein sequence database (Swiss-Prot), and Kyoto Encyclopedia of Genes and Genomes (KEGG), euKaryotic Orthologous Groups (KOG). The expression profiles of candidate gene were obtained from *E. sibiricus* abscission zone transcriptome data (https://www.ncbi.nlm.nih.gov/biosample/6545378). The formula log_2_ (FC) was used to calculate the transcript fold-change, and the false discovery rate (FDR) control method was applied for the correction for multiple tests [27]. Significant differentially expressed transcripts (DETs) between two samples were identified only when an absolute value of the log_2_ (FC) ≥ 2 and FDR significance score ≤ 0.01 were set as the thresholds. A heatmap was constructed for candidate genes using the Heatmap Illustrator (HemI 1.0) program (Beijing Institude of Genomics, CAS, Beijing, China) [74].

## Supplementary information

**Additional file 1: Table S1.** SLAF marker and their locations on each linkage.

**Additional file 2: Table S2.** Phenotypic data of parents and F_2_ population in *E. sibiricus.*

**Additional file 3: Table S3.** Thirty identified candidate genes for seed shattering within six QTL regions based on the functional annotation of barley and wheat genomes.

**Additional file 4: Figure S1.** High-density genetic linkage maps of *E. sibiricus.*

**Additional file 5: Figure S2.** Haplotype map of linkage map.

**Additional file 6: Figure S3.** Heat map of linkage map.

## Data Availability

Raw Illumina sequencing data are available in NCBI SRA: SRX6857199-SRX6857400 (https://www.ncbi.nlm.nih.gov/sra/?term=SLAF-seq+of+Elymus+sibiricus), other datasets generated or analysed during this study are included in this published article and its supplementary files.

## Abbreviations

ABA: Abscisic acid
AL: Awn length
BLAST: Basic local alignment search tool
BTS: Breaking tensile strength
Chr: Chromosome
CIM: Composite interval mapping
COG: Cluster of orthologous groups
CV: Coefficient of variation
DETs: Differentially expressed transcripts
ERF: Ethylene-responsive factors
ETR: Ethylene receptor
FC: Fold change
FDR: False discovery rate
FN: Floret number per spike
GC: Guanine-cytosine
GO: Gene ontology
JA: Jasmonic acid
KEGG: Kyoto encyclopedia of genes and genomes
KOG: Clusters of orthologous groups for eukaryotic complete genomes
LG: Linkage groups
LOD: Logarithm of odds
MAS: Marker-assisted selection
MLOD: The modified logarithm of odds
NGS: Next-generation sequencing
Nr: RefSeq non-redundant protein
PCR: Polymerase chain reaction
Pfam: Protein family
PVE: The percentage of phenotypic variation explained
QTL: Quantitative trait loci
SD: Standard deviation
SL: Spike length
SLAF-seq: Specific-locus amplified fragment sequencing
SNP: Single-nucleotide polymorphism
SS: Seed shattering
SS_C_: Seed shattering rate evaluated by five level of classifications
SS_D_: Seed shattering degree assessed by dropping from a height
SW1: 1000 seed weight
Trv/Tri: SNP type, Trv means transversion, Tri means transition
WS: Width of seed
XTHs: Xyloglucan endotransglucosylase
