# Supplementary figures and images for: Construction of the first high-density genetic linkage map and identification of seed yield-related QTLs and candidate genes in Elymus sibiricus, an important forage grass in Qinghai-Tibet Plateau
Source: BMC Genomics. 2019 Nov 14;20:861. doi: 10.1186/s12864-019-6254-4 (PMC6857239; doi:10.1186/s12864-019-6254-4)

LG01

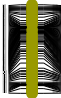

LG02

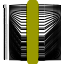

LG03

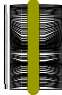

LG04

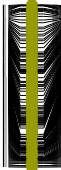

LG05

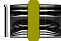

LG06

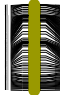

LG07

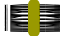

LG08

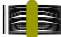

LG09

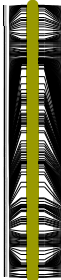

LG10

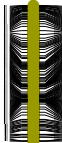

LG11

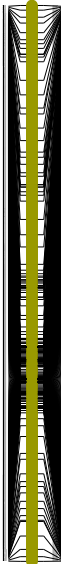

LG12

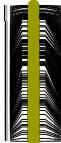

LG13

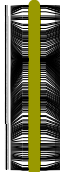

LG14

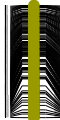

Supplement: Supplementary file 4 — Additional file 4: Figure S1. High-density genetic linkage maps of E. sibiricus. [file 12864_2019_6254_MOESM4_ESM.pdf]
