# Supplementary material for: Construction of the first high-density genetic linkage map and identification of seed yield-related QTLs and candidate genes in Elymus sibiricus, an important forage grass in Qinghai-Tibet Plateau
Source: BMC Genomics. 2019 Nov 14;20:861. doi: 10.1186/s12864-019-6254-4 (PMC6857239; doi:10.1186/s12864-019-6254-4)
Supplement: Supplementary file 5 — Additional file 5: Figure S2. Haplotype map of linkage map. [file 12864_2019_6254_MOESM5_ESM.doc]

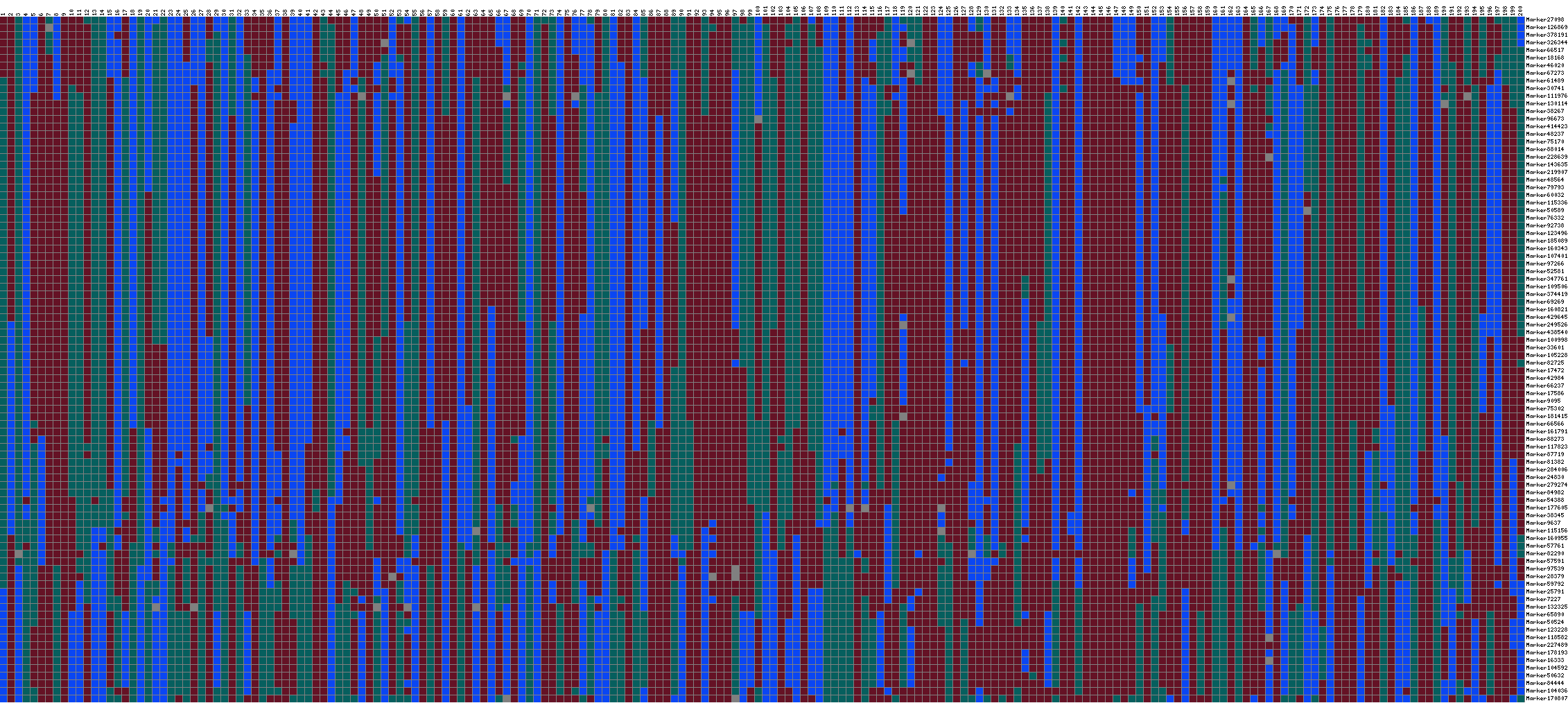


**LG1**


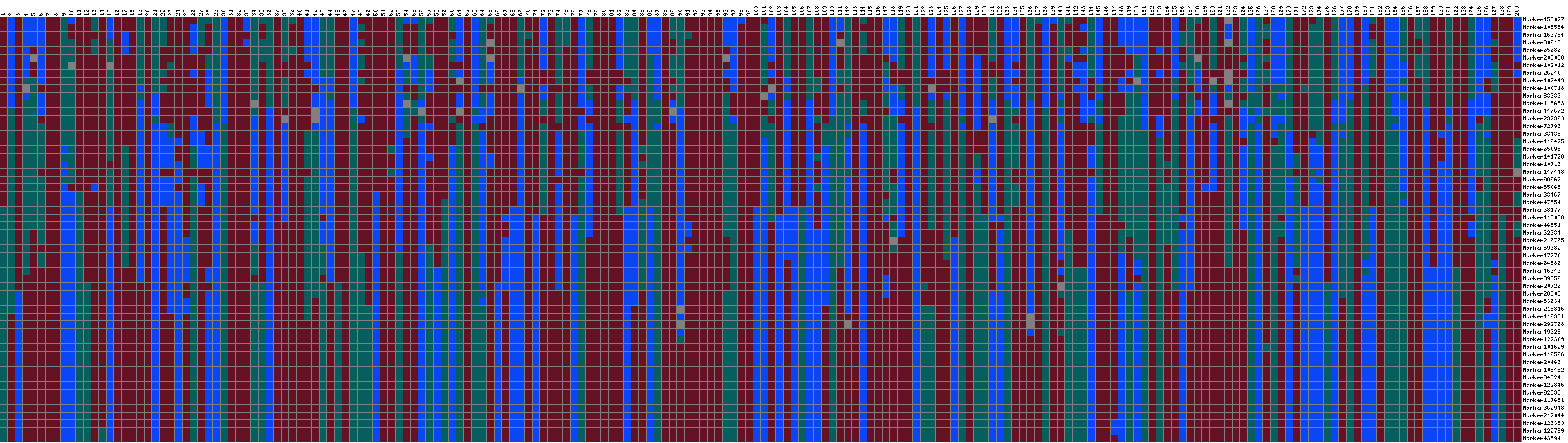


**LG2**

**
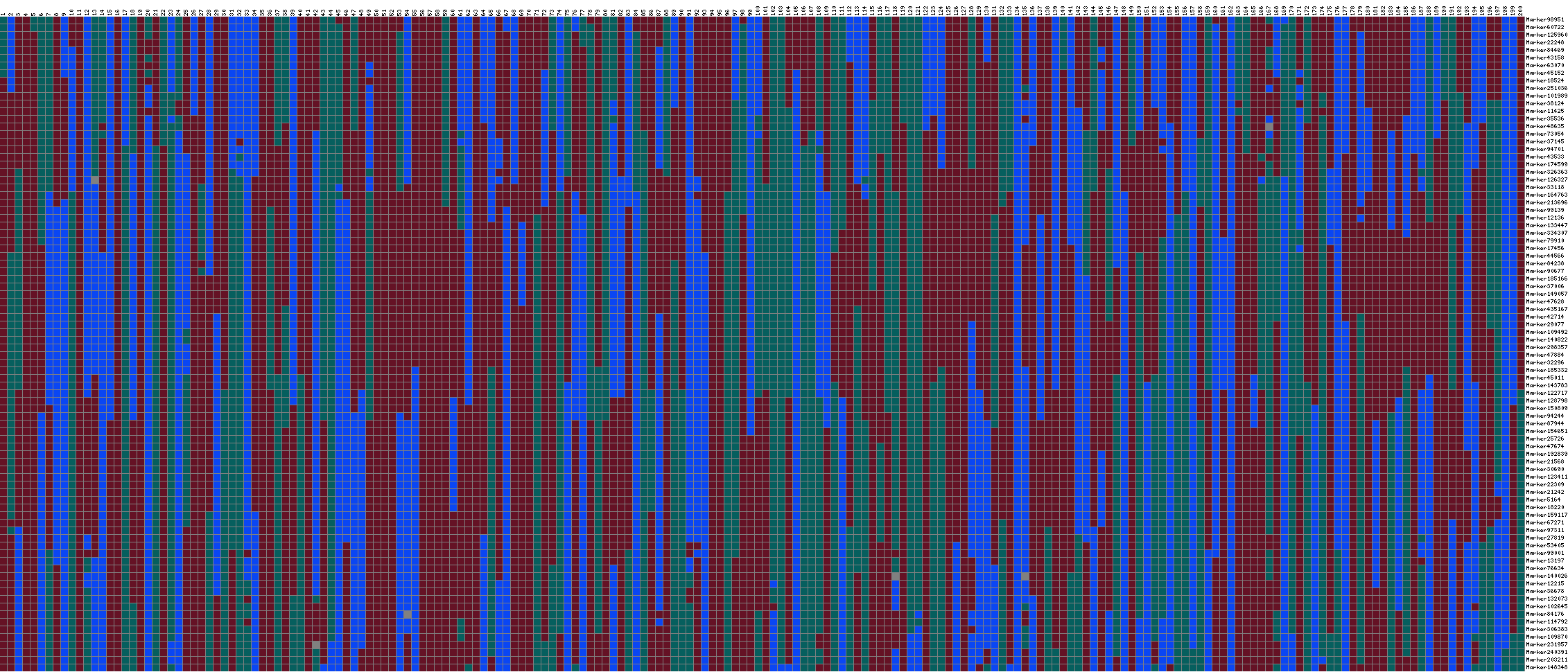
**

**LG3**

**
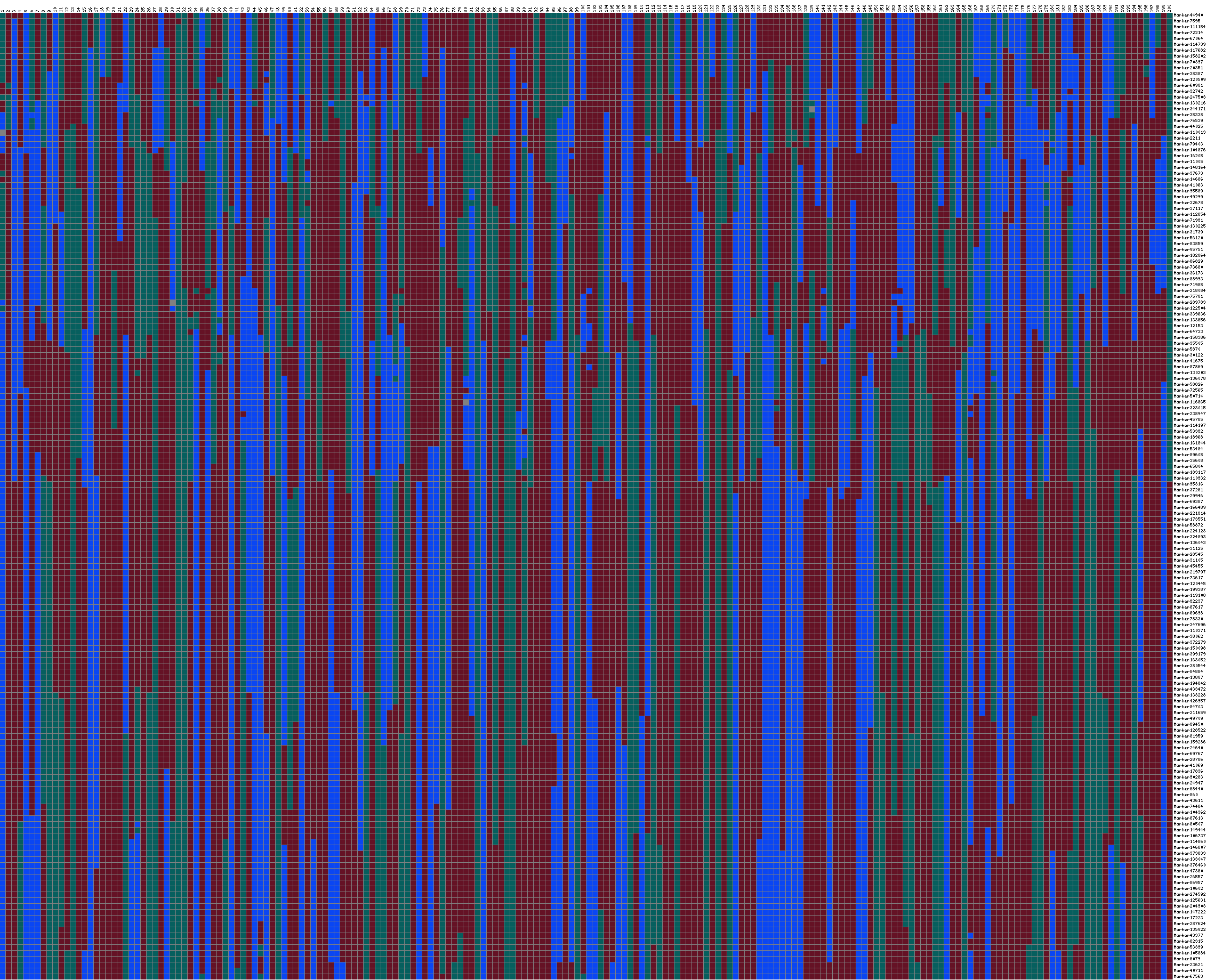
**

**LG4**

**
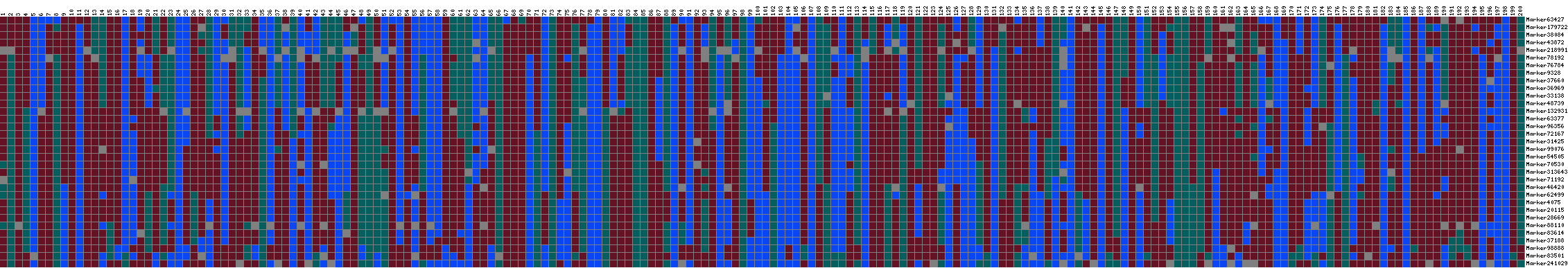
**

**LG5**

**
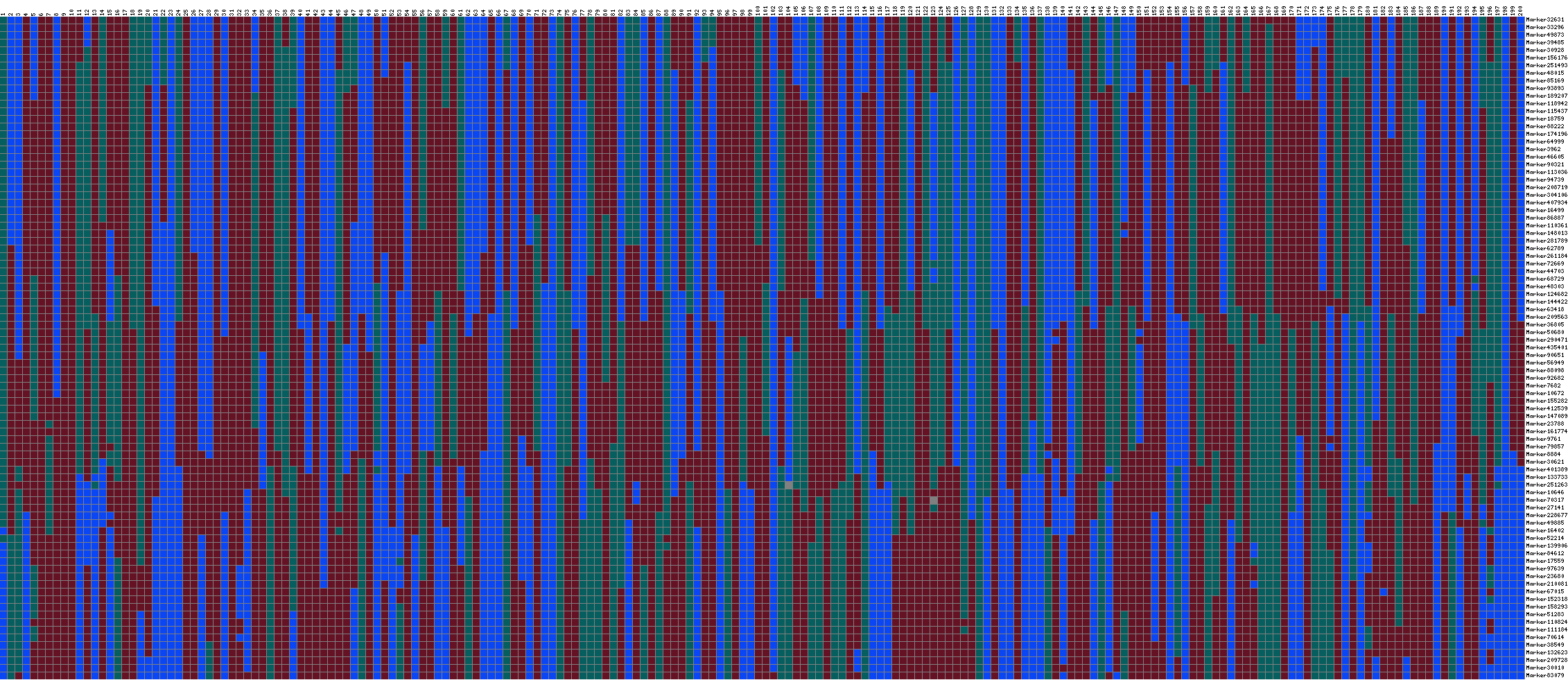
**

**LG6**

**
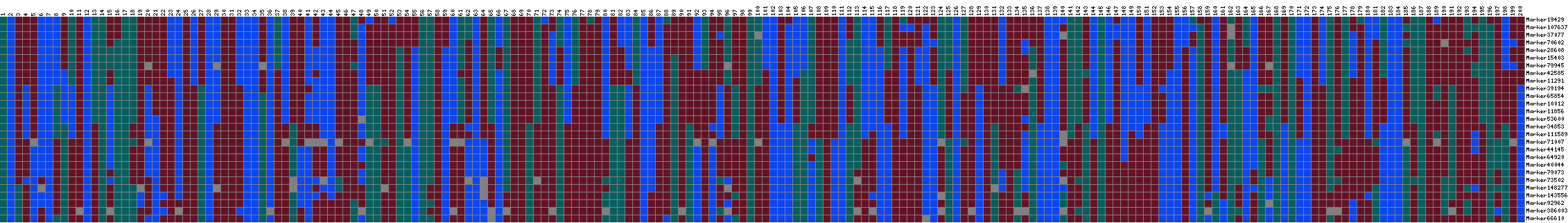
**

**LG7**

**
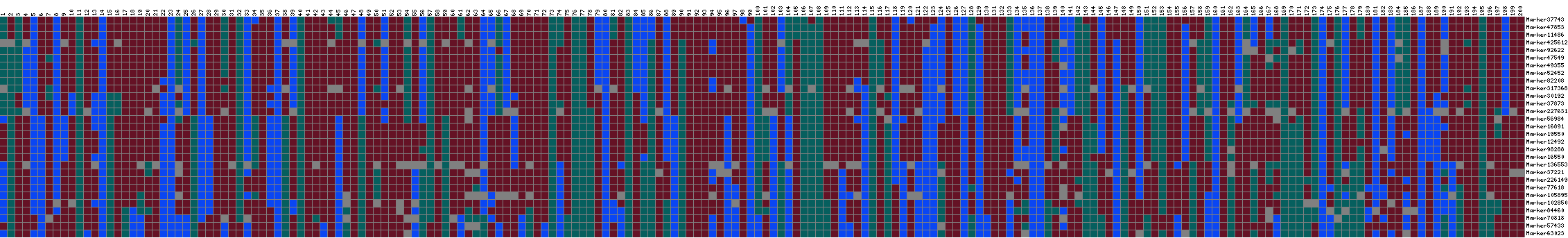
**

**LG8**

**
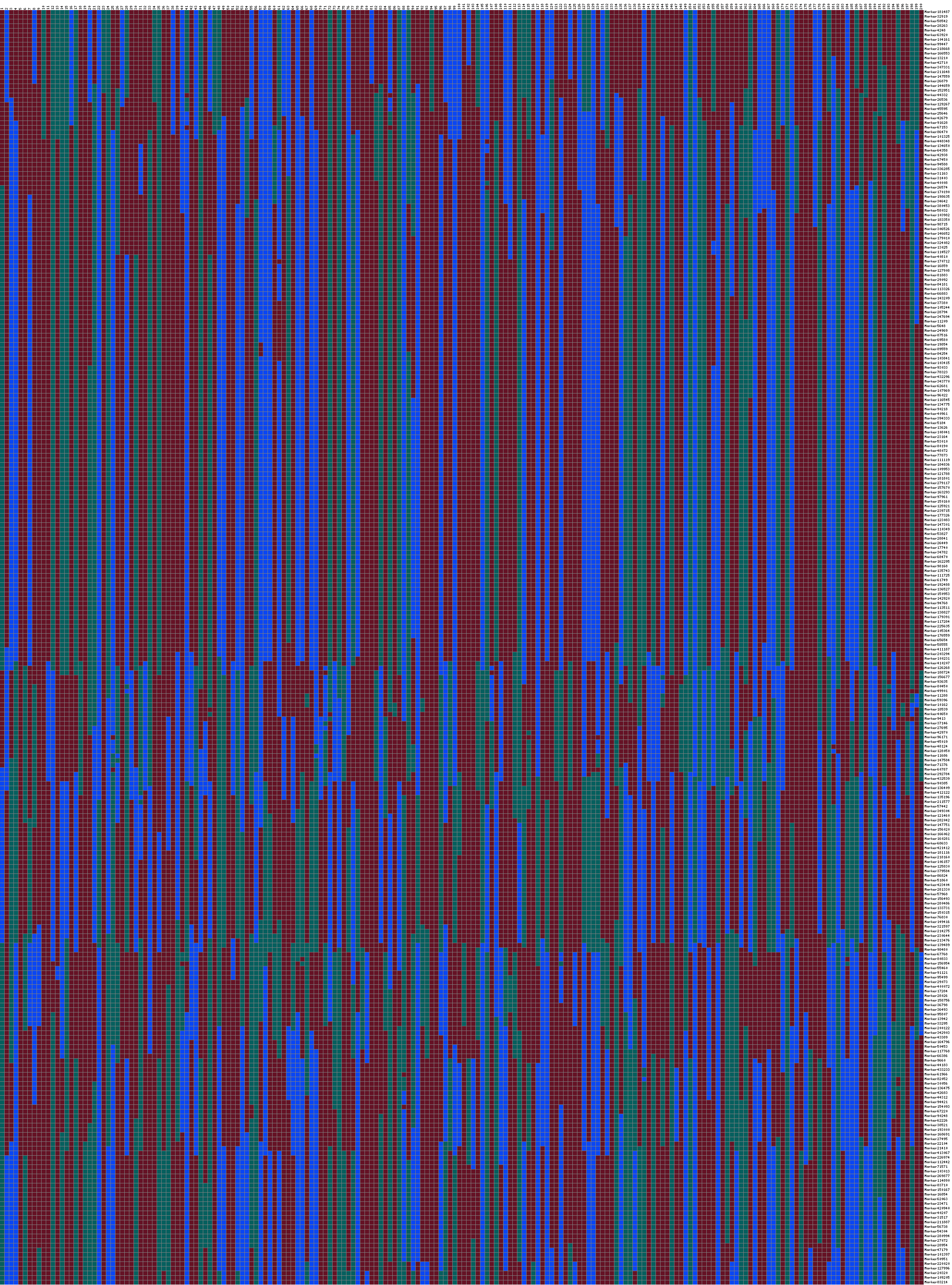
**

**LG9**

**
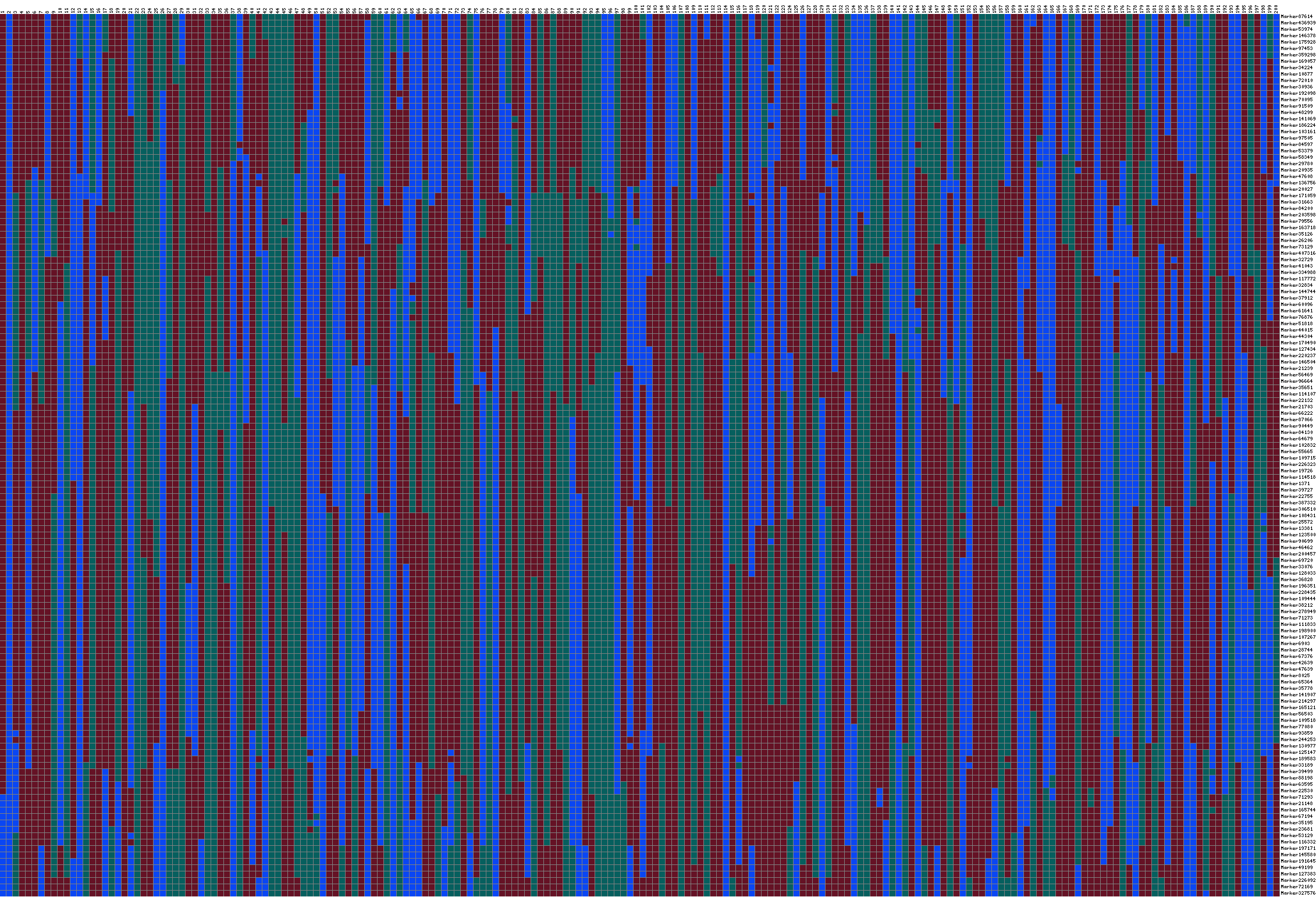
**

**LG10**

**
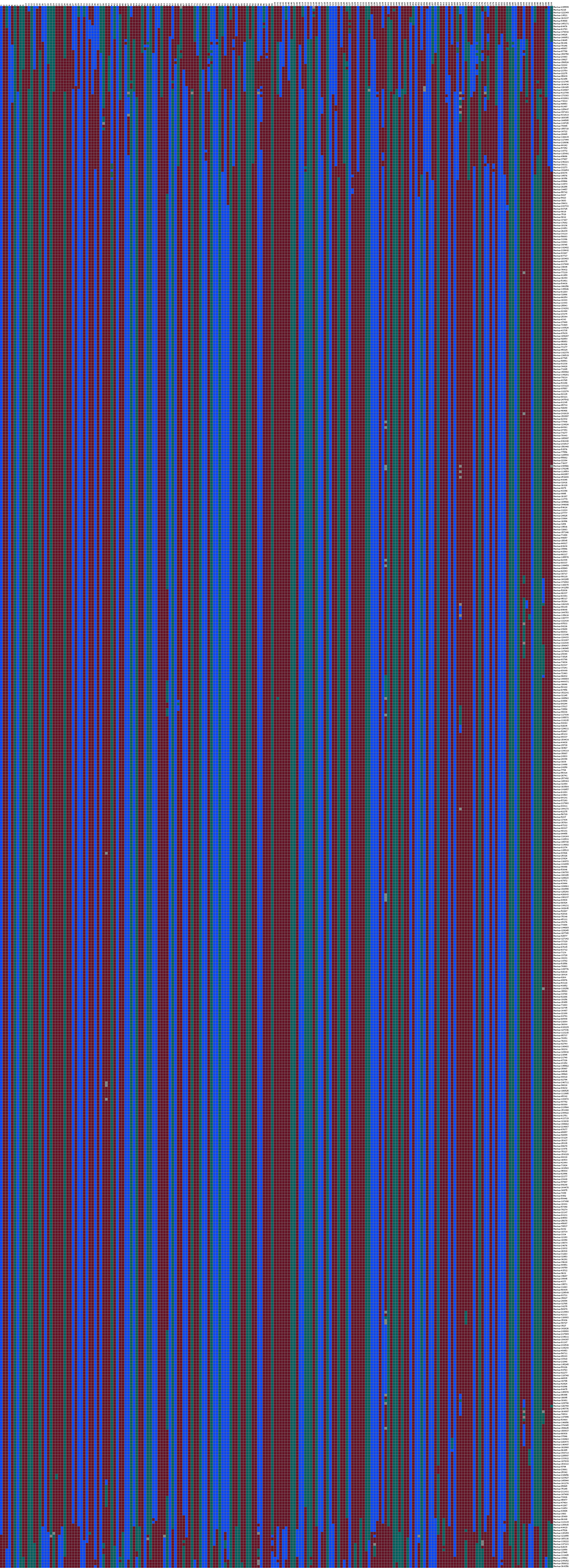
**

**LG11**

**
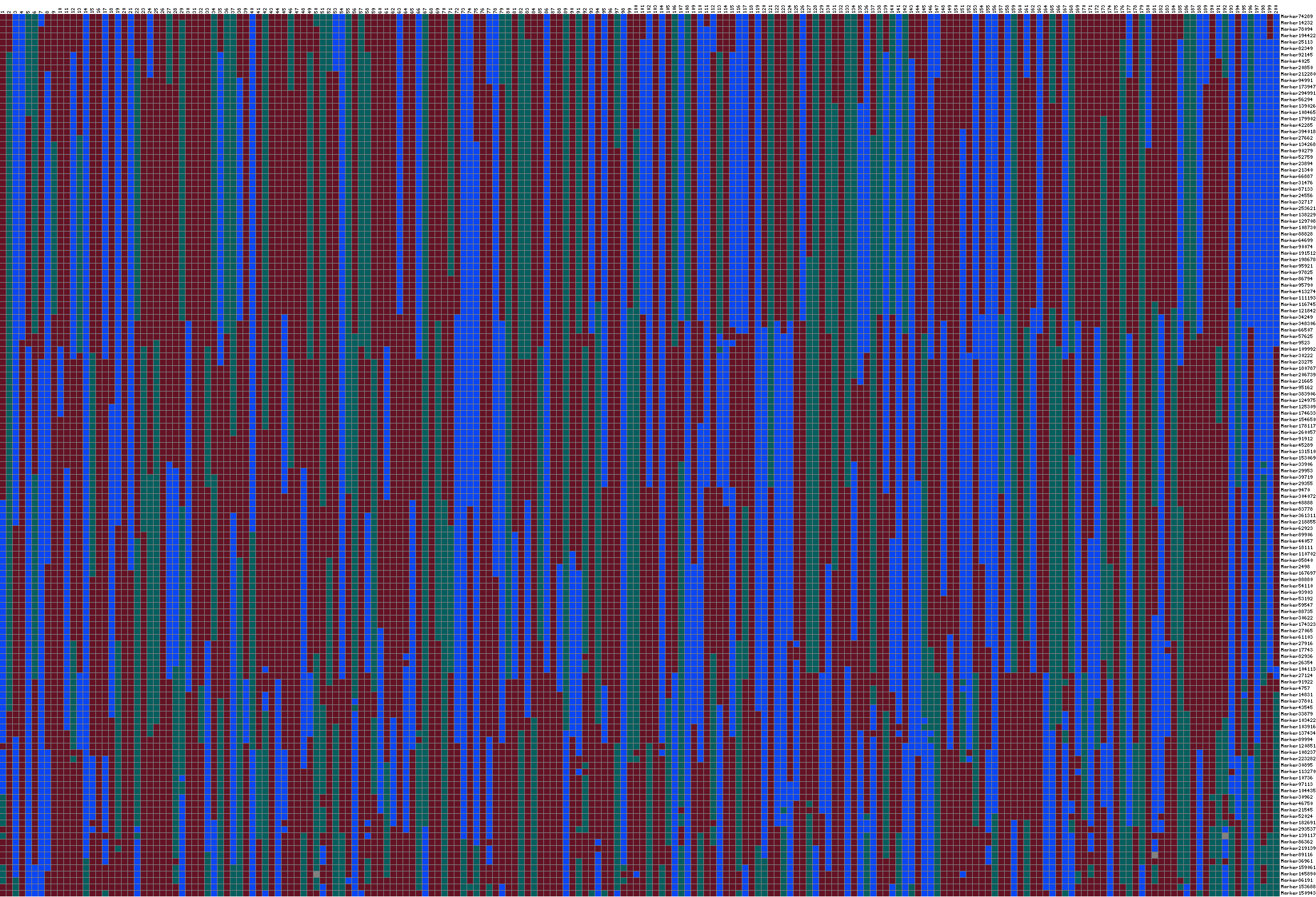
**

**LG12**

**
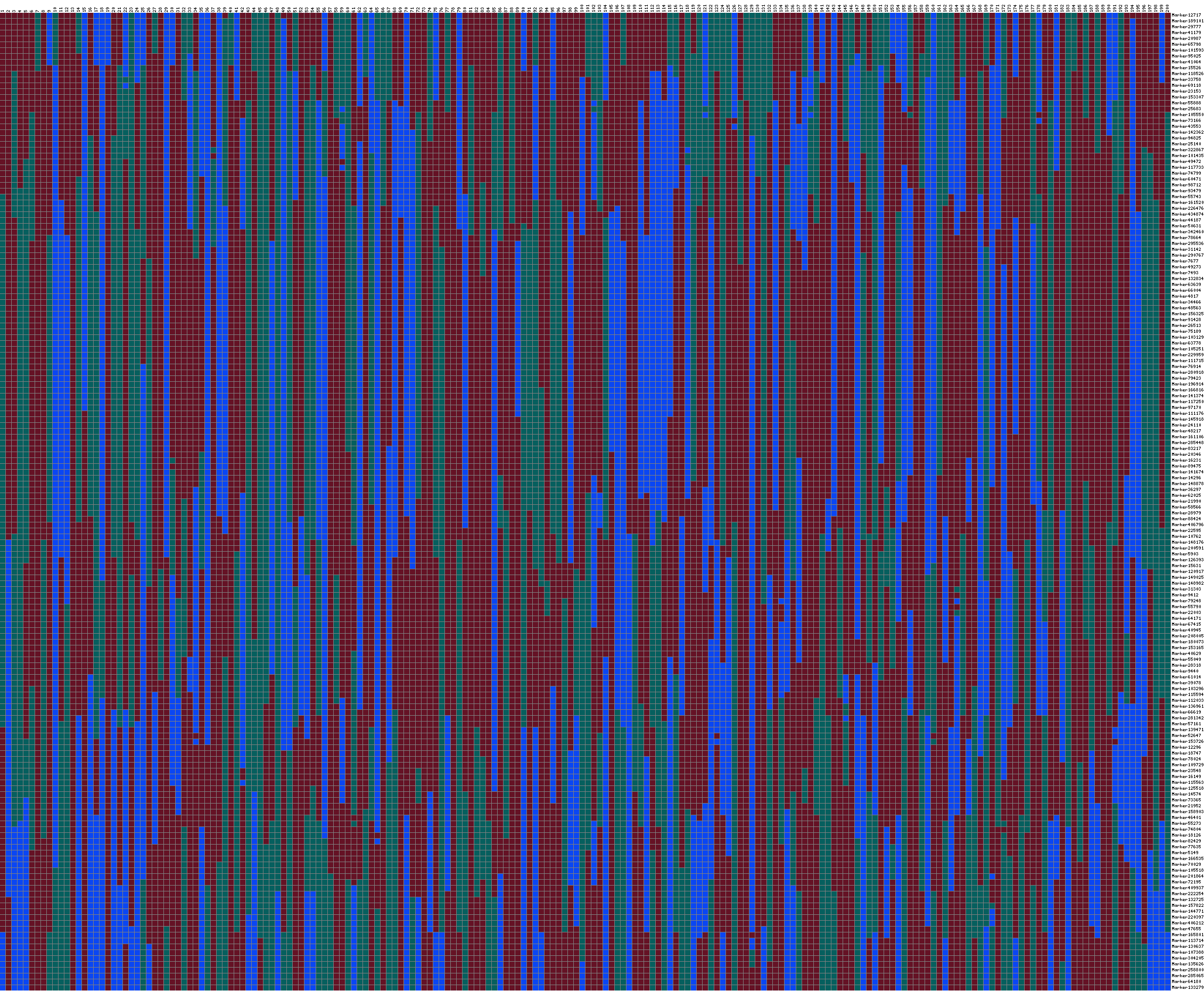
**

**LG13**

**
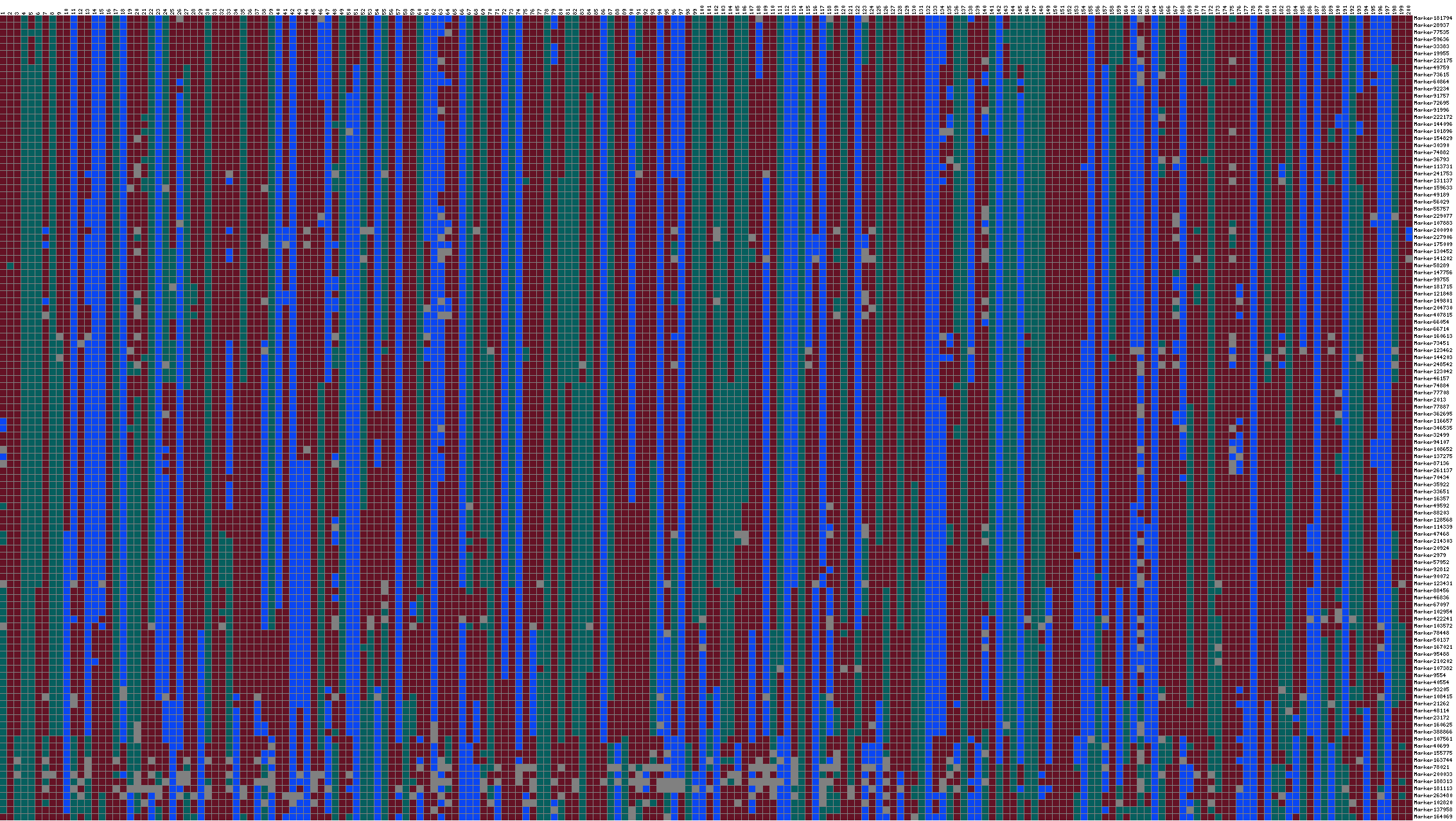
**

**LG14**

**Figure S2. Haplotype map of linkage map.** Each row represents a mapped marker. Markers are ranked in accordance with the map order. Each of the two columns represents an individual; blank columns are used between two individuals. The first columns represents the paternal chromosomes, and second columns represents maternal chromosomes. The green and blue regions in the columns represent the first and second alleles from the parents, respectively. The white column represents the source of alleles that cannot be judged. The gray region represent the deleted alleles.
