# Supplementary material for: Construction of the first high-density genetic linkage map and identification of seed yield-related QTLs and candidate genes in Elymus sibiricus, an important forage grass in Qinghai-Tibet Plateau
Source: BMC Genomics. 2019 Nov 14;20:861. doi: 10.1186/s12864-019-6254-4 (PMC6857239; doi:10.1186/s12864-019-6254-4)
Supplement: Supplementary file 6 — Additional file 6: Figure S3. Heat map of linkage map. [file 12864_2019_6254_MOESM6_ESM.doc]

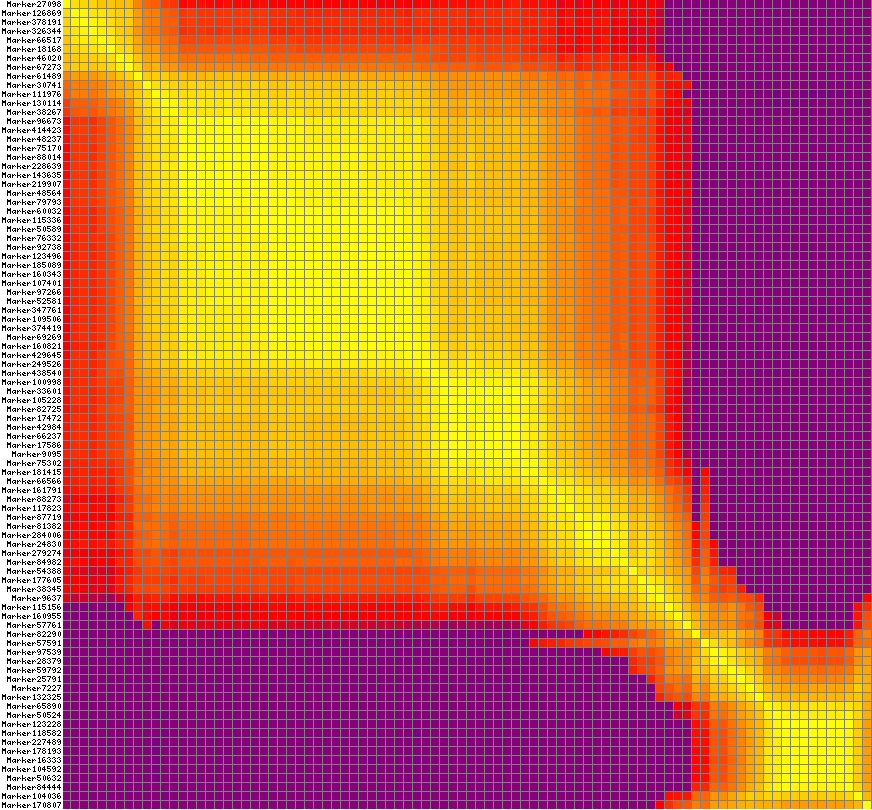


**LG1**


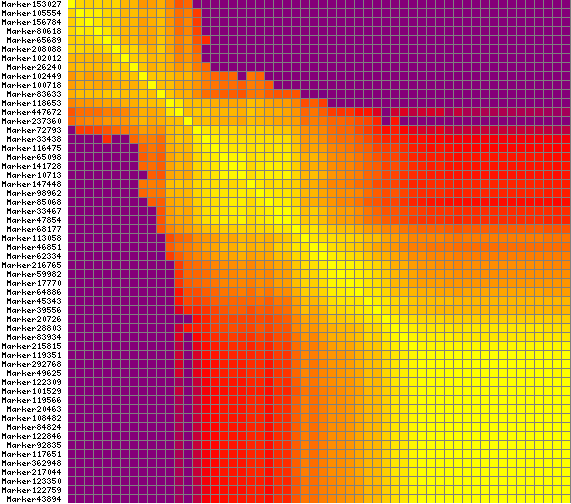


**LG2**


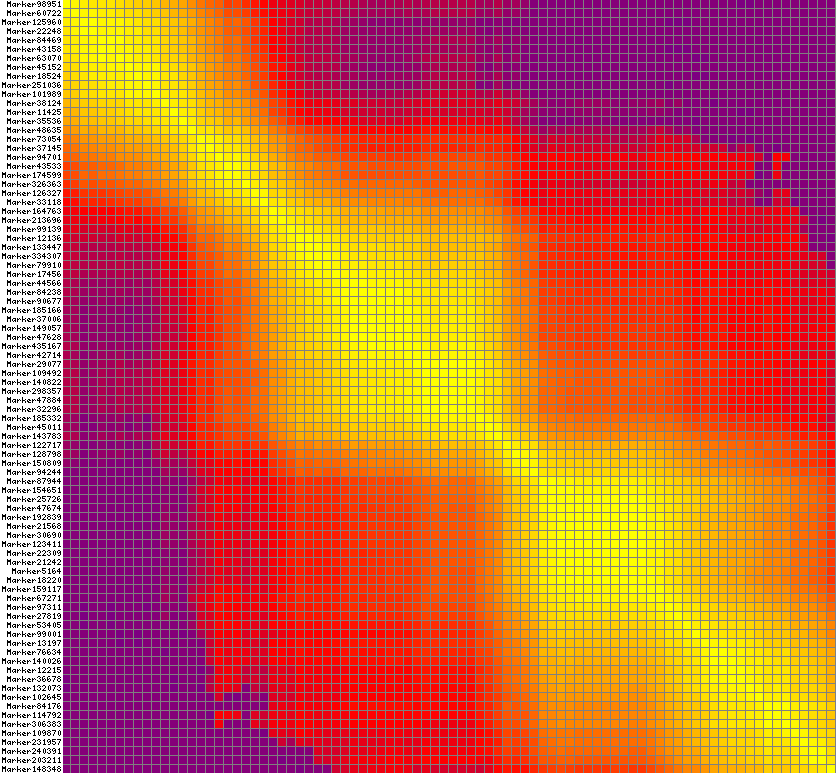


**LG3**


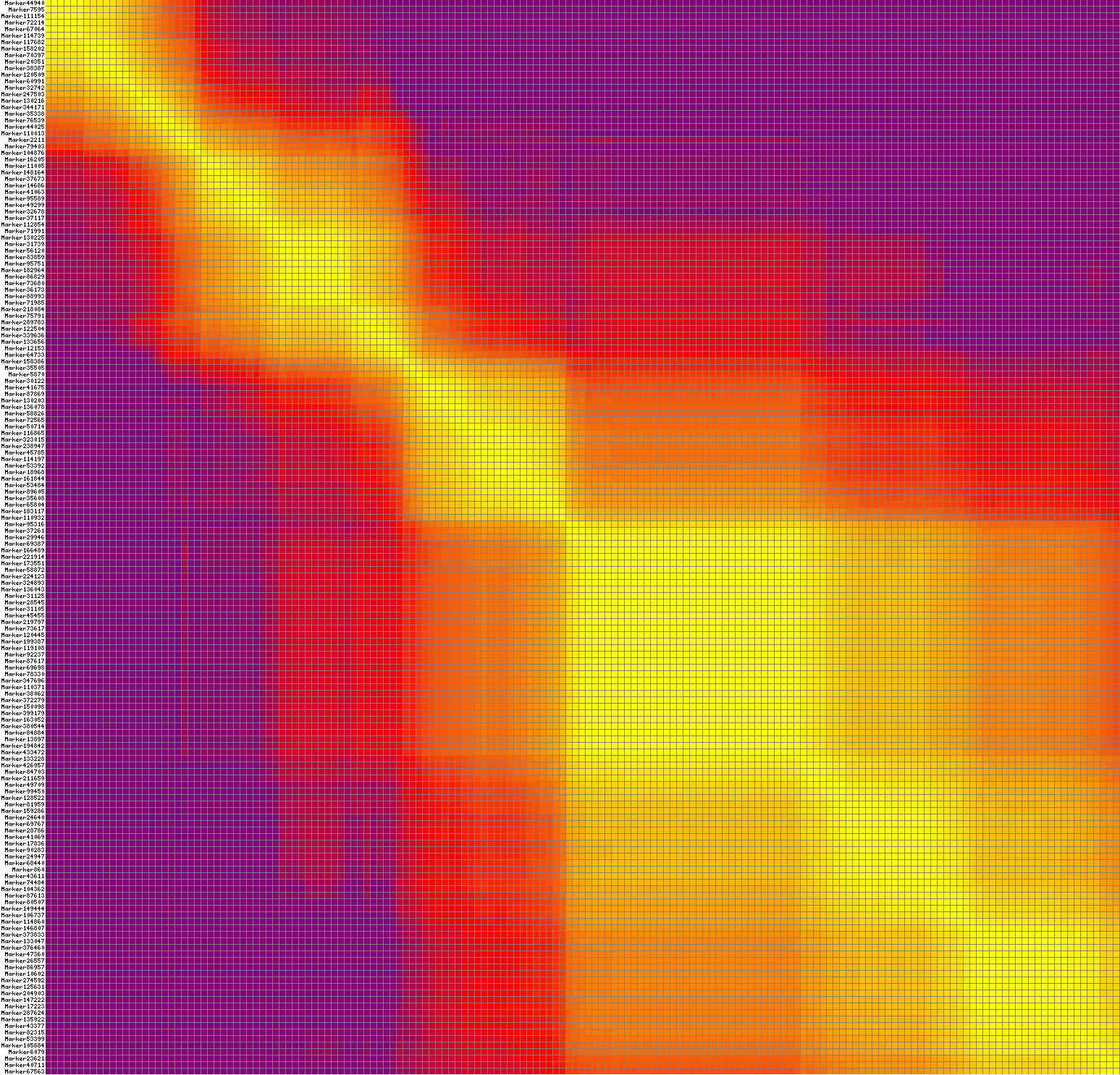


**LG4**


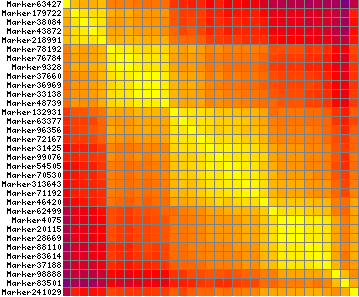


**LG5**


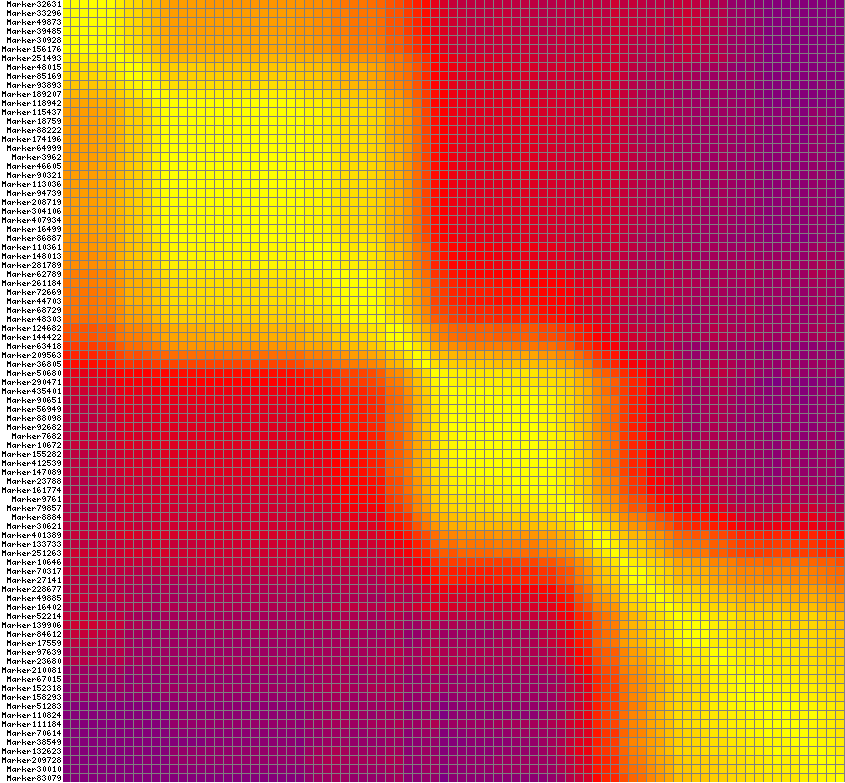


**LG6**


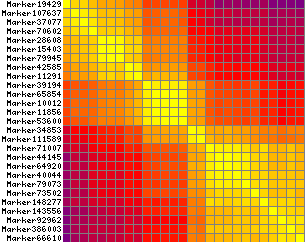


**LG7**


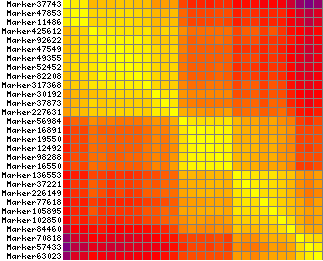


**LG8**


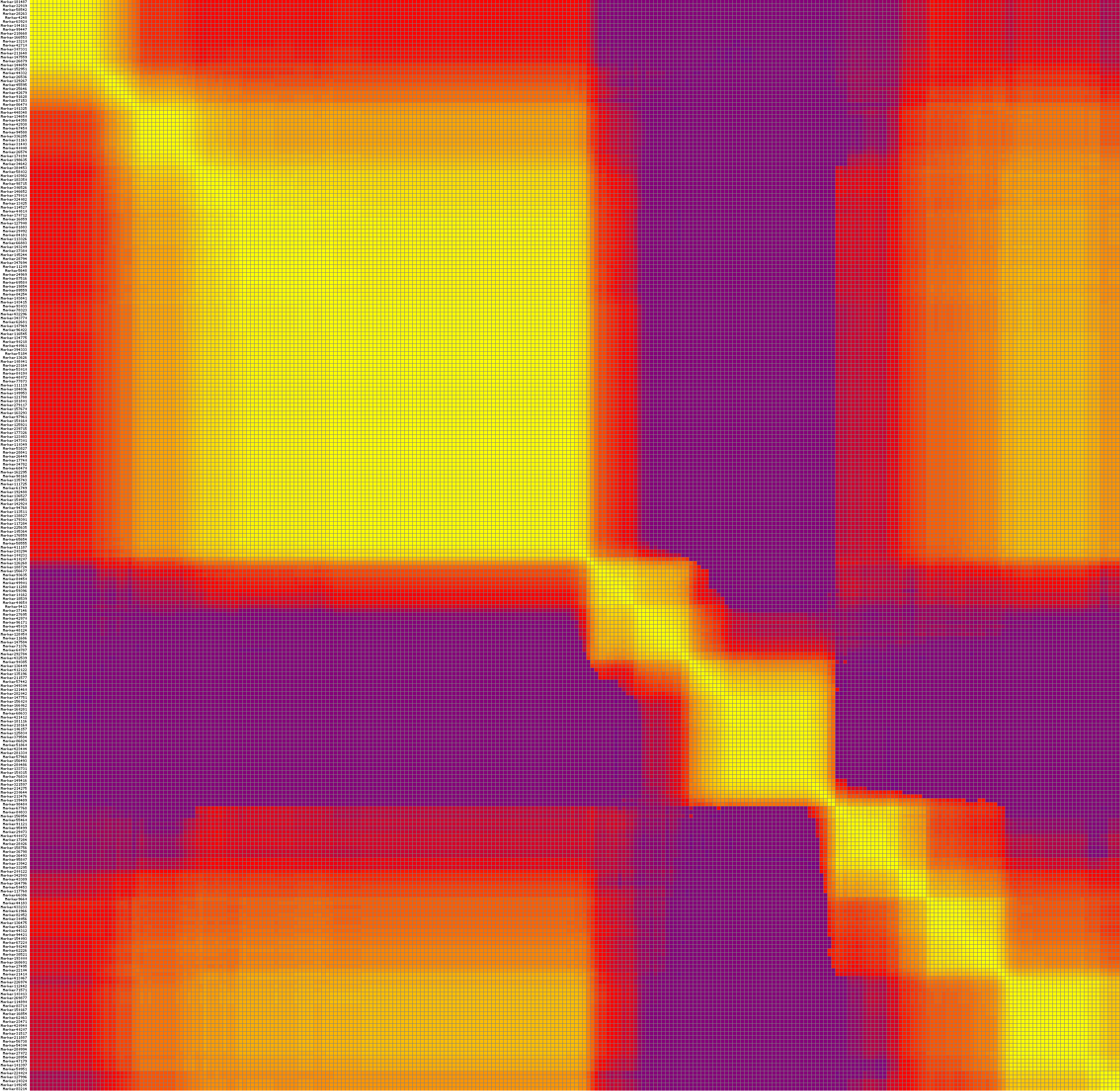


**LG9**


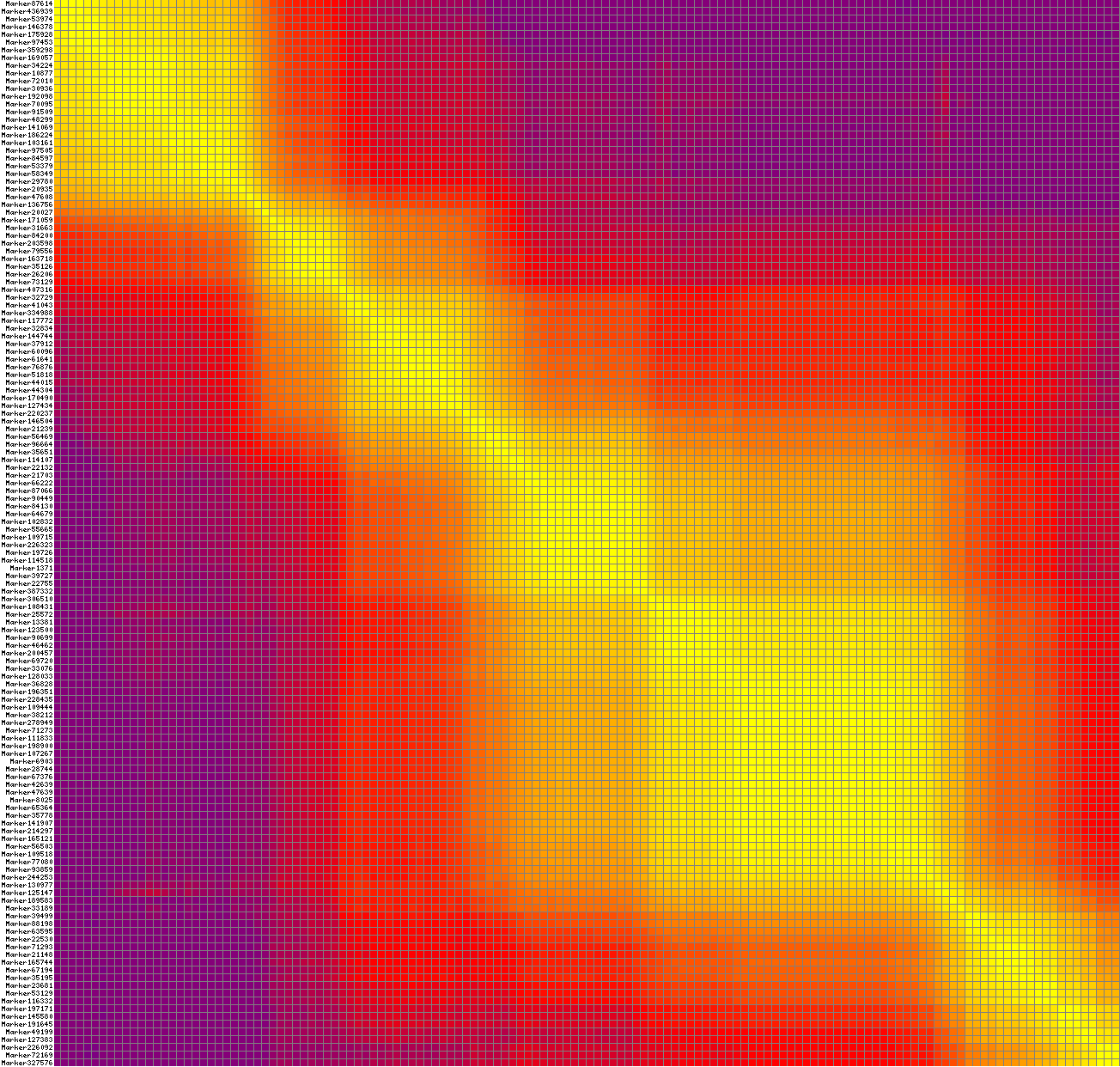


**LG10**


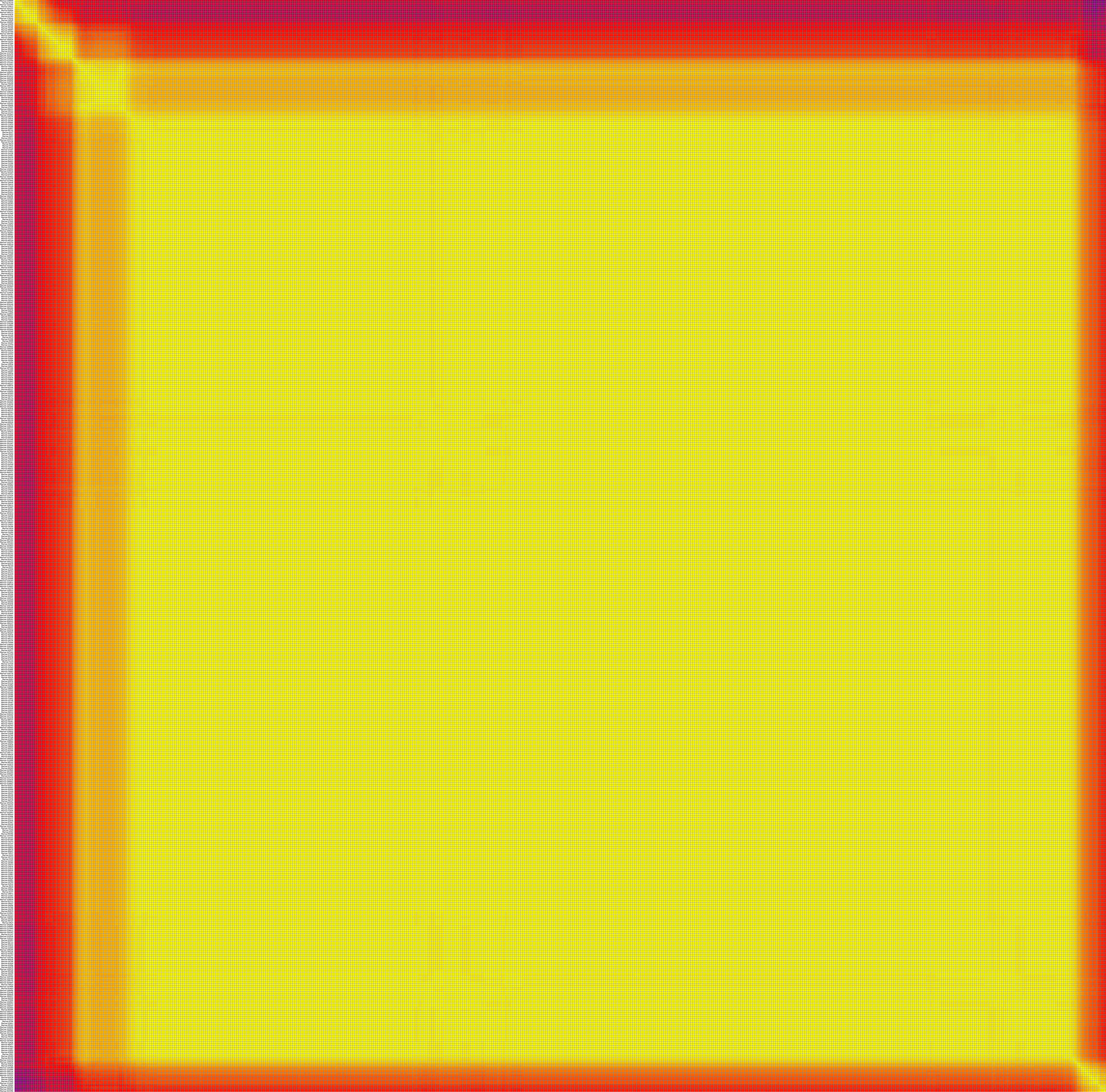


**LG11**


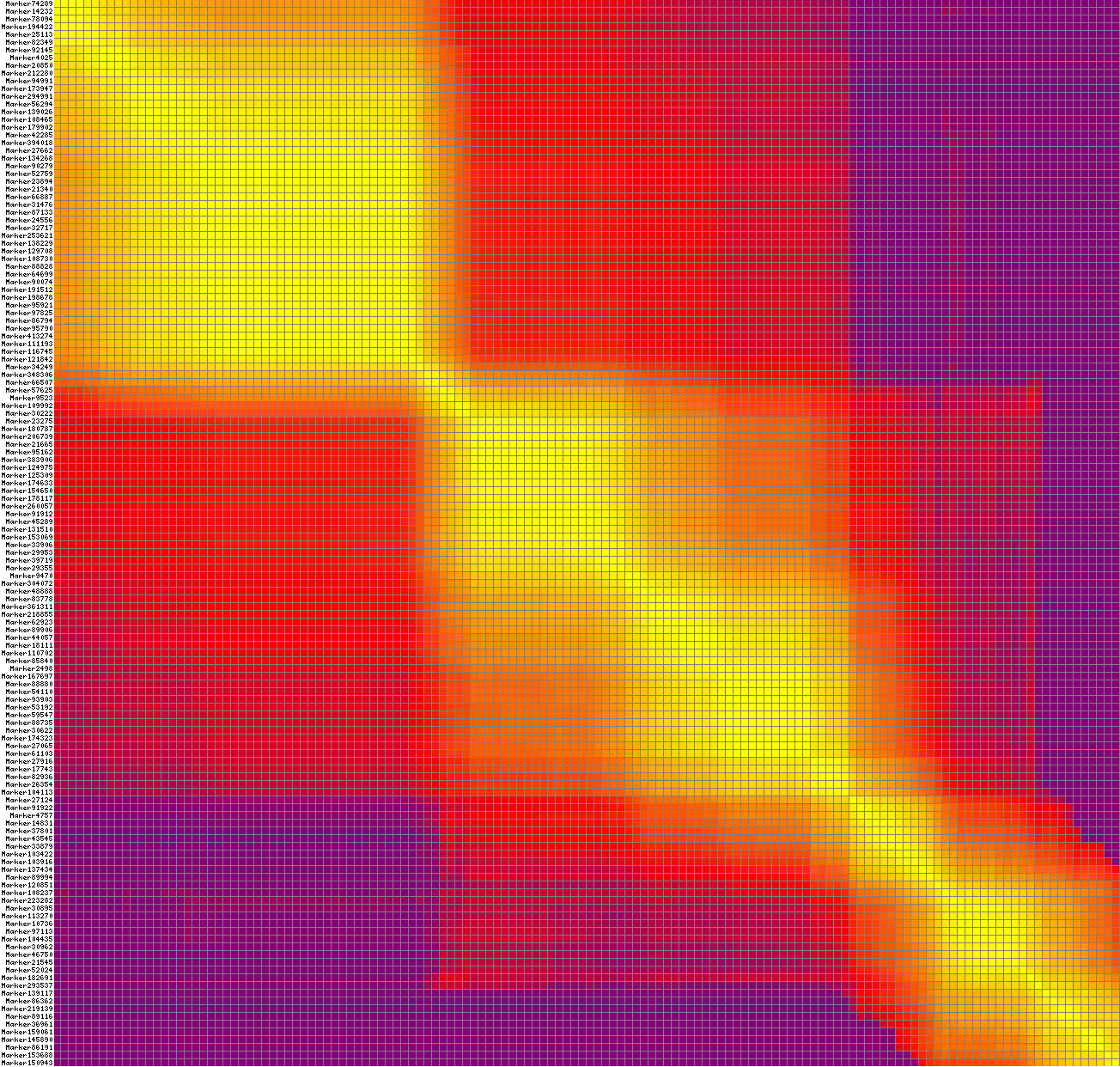


**LG12**


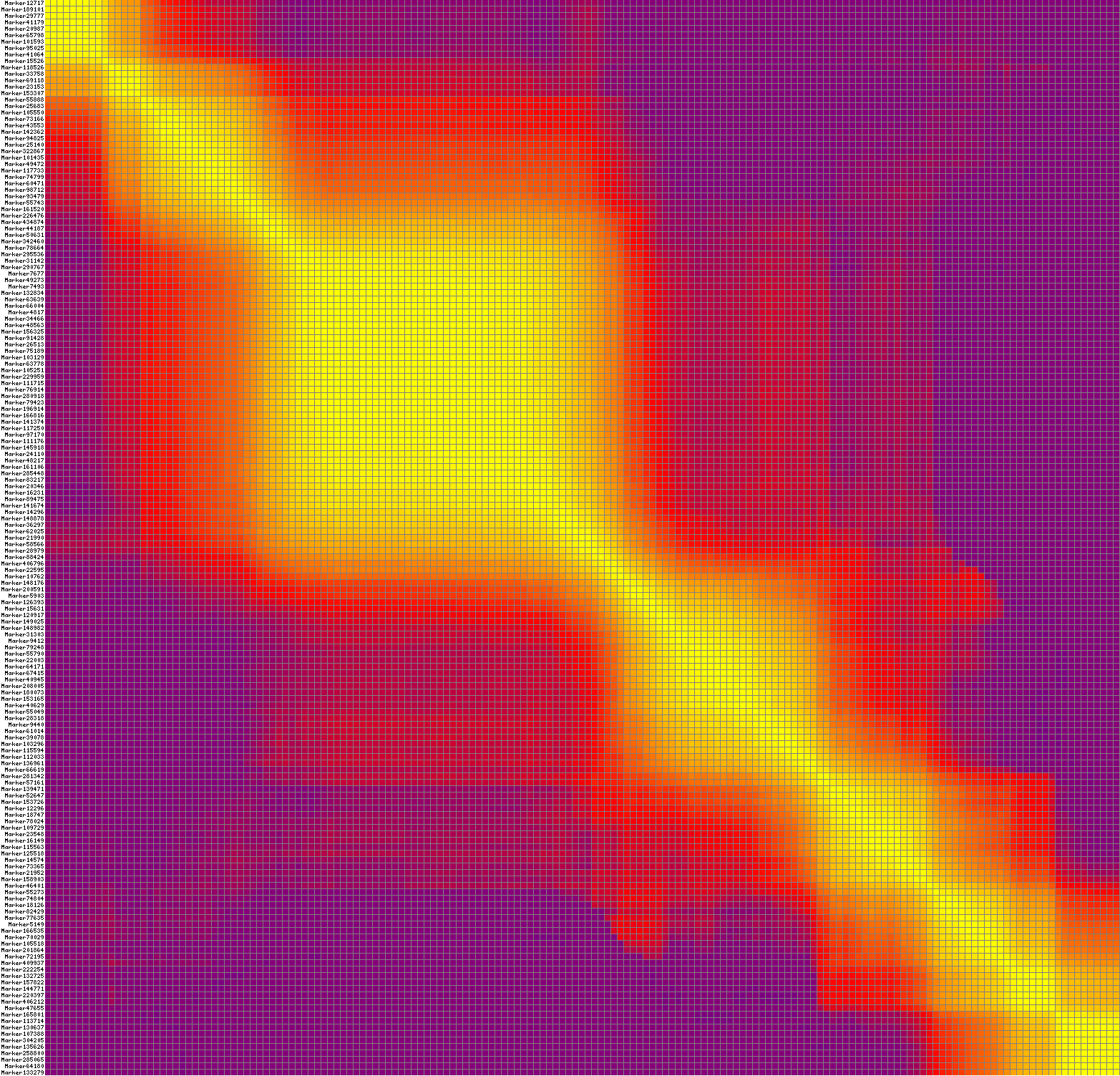


**LG13**


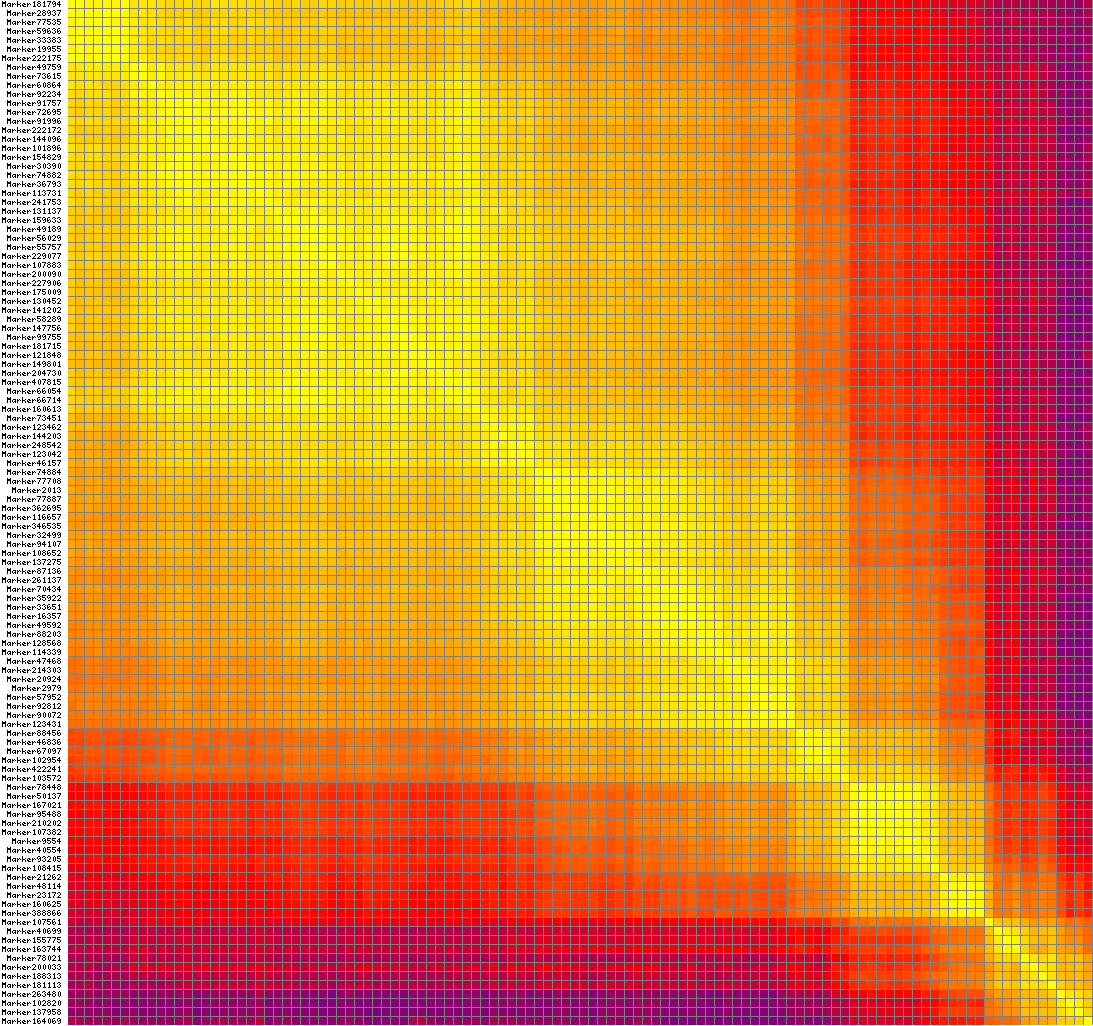


**LG14**

**Figure S3. Heat map of the linkage maps**. Markers of each row and column are ranked according to the map order; each small square represents the recombination rate (r) between the two markers. Color from yellow to purple means the r value from large to small. The closer between two markers the smaller the recombination rata.
